# Targeting cellular senescence with Traditional Chinese Medicine: advances in neurodegenerative disorder treatment

**DOI:** 10.3389/fneur.2026.1875385

**Published:** 2026-08-19

**Authors:** Huanyue Hai, Muhan Li, Xue Hu, Jingjing Peng, Zhaoxia He

**Affiliations:** 1 Department of Rheumatology and Immunology, General Hospital of Western Theater Command, Chengdu, Sichuan, China; 2 Department of Oncology, General Hospital of Western Theater Command, Chengdu, Sichuan, China

**Keywords:** active components, cellular senescence, neurodegenerative diseases, signaling pathways, therapeutic potential, Traditional Chinese Medicine

## Abstract

Neurodegenerative diseases (NDDs), including Alzheimer’s disease (AD), Parkinson’s disease (PD), and Huntington’s disease (HD), represent formidable health challenges, particularly within aging populations. Cellular senescence, marked by irreversible cell cycle arrest and the secretion of pro-inflammatory cytokines, has emerged as a crucial factor in the pathogenesis of these disorders. The accumulation of senescent cells in the nervous system exacerbates neuroinflammation, impairs neuronal function, and accelerates tissue degeneration, all of which are key features of NDDs. In this context, bioactive components derived from Traditional Chinese Medicine (TCM) have attracted significant attention for their potential to modulate cellular senescence and alleviate neurodegeneration. This review examines the shared molecular signaling pathways linking cellular senescence to NDDs, explores the mechanisms by which TCM-derived compounds—such as ginsenosides, ginkgo biloba extract, and curcumin—intervene in senescence-related processes, and discusses the translational potential of these natural compounds for clinical application.

## 1 Introduction

Neurodegenerative diseases (NDDs) represent a heterogeneous group of chronic, progressive disorders of the central nervous system, frequently associated with aging (1, 2). Prominent examples of these conditions include Alzheimer’s disease (AD) and Parkinson’s disease (PD), which are characterized by the gradual loss of neuronal structure and function. While the pathophysiology of NDDs is multifactorial, increasing evidence suggests that cellular senescence plays a pivotal role in their progression (3, 4). Cellular senescence is defined as an irreversible cessation of the cell cycle, which contributes to tissue aging, persistent low-grade inflammation, and a decline in organ function—pathological processes that are fundamental to the onset and exacerbation of neurodegeneration (5, 6).

In this context, Traditional Chinese Medicine (TCM), which has a long history of use in treating age-related disorders, offers a promising source of natural compounds with neuroprotective and anti-senescence properties (7, 8). Indeed, several TCM-derived agents have already entered clinical practice for neurological indications. Huperzine A, an alkaloid isolated from *Huperzia serrata*, is approved in China for the symptomatic treatment of AD (9); standardized ginkgo biloba extract is widely used for cognitive impairment and has been evaluated in large randomized controlled trials (10); and ginseng preparations remain among the most frequently used remedies for age-related cognitive and motor decline (11, 12). Modern pharmacological studies have shown that these agents converge on aging-relevant targets—including mitochondrial homeostasis, oxidative stress, and neuroinflammation—providing a mechanistic rationale for re-examining TCM through the lens of cellular senescence. Many bioactive constituents derived from TCM have been shown to mitigate cellular senescence and promote neuronal survival (11, 12). These compounds act through various molecular mechanisms, modulating key signaling pathways involved in cell cycle regulation, oxidative stress, and inflammatory responses. This review aims to critically evaluate the molecular mechanisms through which TCM-derived bioactive molecules influence cellular senescence and neurodegeneration. Furthermore, it will highlight their potential therapeutic applications in delaying or alleviating the progression of NDDs, with a focus on the translational potential of these compounds in clinical settings.

## 2 Key signaling pathways linking cellular senescence to neurodegenerative diseases

### 2.1 Roles of cellular senescence in neurodegenerative diseases

Cellular senescence has emerged as a central biological mechanism driving the pathogenesis of major neurodegenerative diseases, including AD, PD, and HD. These disorders share common clinical hallmarks of progressive neurological decline, yet exhibit distinct pathological features: AD is characterized by progressive memory loss and cognitive impairment, primarily driven by amyloid-β (Aβ) plaque deposition and neurofibrillary tangles composed of hyperphosphorylated tau protein (1); PD manifests with motor symptoms including bradykinesia, resting tremor, and rigidity, resulting from dopaminergic neuron loss in the substantia nigra and *α*-synuclein aggregation (13, 14); while HD presents with choreiform movements and cognitive decline due to mutant huntingtin (mHTT)-mediated striatal neurodegeneration (15, 16).

Although these disorders converge on common senescence hallmarks—most notably the SASP, irreversible cell cycle arrest, and mitochondrial dysfunction—the upstream triggers that initiate senescence, the specific neuronal and glial populations predominantly affected, and the downstream pathological consequences of senescent cell accumulation differ substantially across AD, PD, and HD (5, 6). These disease-specific differences in senescence induction and propagation justify a compartmentalized discussion, as they carry direct implications for targeted therapeutic strategies.

Critically, cellular senescence creates a vicious cycle in these diseases through multiple interconnected mechanisms (3). Senescent cells develop a characteristic senescence-associated secretory phenotype (SASP), releasing pro-inflammatory cytokines (IL-6, TNF-*α*), matrix metalloproteinases (MMPs), and reactive oxygen species (ROS) that perpetuate chronic neuroinflammation (17, 18). In AD, senescent astrocytes and microglia promote Aβ accumulation through impaired clearance mechanisms while simultaneously activating tau-phosphorylating kinases like GSK-3β (19, 20); in PD, senescence-induced lysosomal dysfunction exacerbates *α*-synuclein aggregation, and in HD, senescent microglia exhibit sustained NLRP3 inflammasome activation that amplifies mHTT toxicity. Notably, recent breakthroughs demonstrate that selective elimination of senescent cells can attenuate key pathological features - clearing p16-positive glial cells reduces tau pathology, senolytic treatment improves Aβ clearance, and targeting SASP components mitigates *α*-synuclein spread. These findings offer novel therapeutic opportunities for multiple neurological disorders.

#### 2.1.1 Alzheimer’s disease (AD)

In Alzheimer’s disease, the accumulation of senescent astrocytes, microglia, and endothelial cells contributes to a chronic inflammatory environment that accelerates cognitive decline. Senescent astrocytes release a variety of pro-inflammatory factors, including IL-6, IL-1β, and TNF-*α*, which contribute to neuroinflammation and neuronal damage (21–24). Recent studies have shown that oxidative stress-induced senescence in astrocytes is linked to transcriptomic alterations that exacerbate neurodegeneration and impair synaptic function (25, 26). Targeting senescent cells has emerged as a promising therapeutic strategy to reduce neuroinflammation and improve cognitive function in Alzheimer’s disease (19, 20). Furthermore, recent interventions targeting the SASP in senescent astrocytes have shown potential in reducing neuroinflammation and mitigating the progression of Alzheimer’s disease (4, 27).

Notably, the senescent cell burden may track AD severity: glial senescence markers increase in parallel with tau pathology in post-mortem brains (4, 19), and peripheral senescence-associated signaling (e.g., lymphocyte p-mTOR) correlates with memory decline in living patients (28), although prospective validation is still needed.

#### 2.1.2 Parkinson’s disease (PD)

Parkinson’s disease is marked by the accumulation of *α*-synuclein aggregates, mitochondrial dysfunction, and oxidative stress, all of which contribute to the formation of senescent neurons and glial cells (29–31). In this context, senescent astrocytes and microglia fail to clear α-synuclein aggregates, leading to their accumulation and further exacerbating neurodegeneration. The accumulation of toxic proteins induces neuroinflammation and impairs dopaminergic function, resulting in motor deficits (29, 30, 32). Beyond impaired *α*-synuclein clearance, senescence also undermines dopamine homeostasis, as mitochondrial dysfunction and oxidative stress reduce tyrosine hydroxylase activity and dopamine synthesis in surviving nigral neurons (13, 29). Moreover, advanced glycation end products (AGEs) accumulated with aging promote *α*-synuclein cross-linking and RAGE-dependent microglial activation (33), and the familial PD gene product DJ-1 (PARK7) detoxifies the AGE precursor methylglyoxal, genetically linking glycation stress to PD pathogenesis (34). In animal models of Parkinson’s disease, targeting senescent glial cells has been shown to reduce *α*-synuclein deposition and improve motor function (13). Consequently, the senescence pathways associated with neurodegeneration have become emerging therapeutic targets for Parkinson’s disease, offering potential for alleviating the motor symptoms associated with the disease (35–37).

#### 2.1.3 Huntington’s disease (HD)

In Huntington’s disease, mitochondrial dysfunction, oxidative stress, and the accumulation of mutant huntingtin protein drive neuronal senescence and neurodegeneration. Senescent neurons in the striatum exhibit impaired autophagy and accumulate toxic protein aggregates, disrupting cellular homeostasis and promoting neurodegeneration (15, 16, 38). Impaired clearance of toxic proteins in senescent neurons contributes to the progressive nature of Huntington’s disease (39). Regarding the interplay between senescence and the expanded CAG repeat itself, current evidence supports a bidirectional relationship. While the inherited CAG length is fixed, its somatic expansion in striatal neurons—driven by age-related DNA damage and mismatch repair—progresses with age and predicts earlier onset (40); in turn, mHTT-driven oxidative stress and DNA damage fuel neuronal senescence, forming a self-reinforcing loop. Recent research suggests that targeting the mammalian target of rapamycin (mTOR) pathway and activating autophagy could help reduce protein aggregation and protect neurons in Huntington’s disease models (15, 41, 42). Furthermore, activation of AMPK has been shown to improve mitochondrial function and reduce neuronal death in Huntington’s disease (43, 44). These findings underscore the therapeutic potential of targeting senescence-associated pathways for the treatment of Huntington’s disease (45).

### 2.2 Signaling pathways regulating cellular senescence associated with neurodegenerative diseases

#### 2.2.1 mTOR signaling pathway

**Figure 1:**
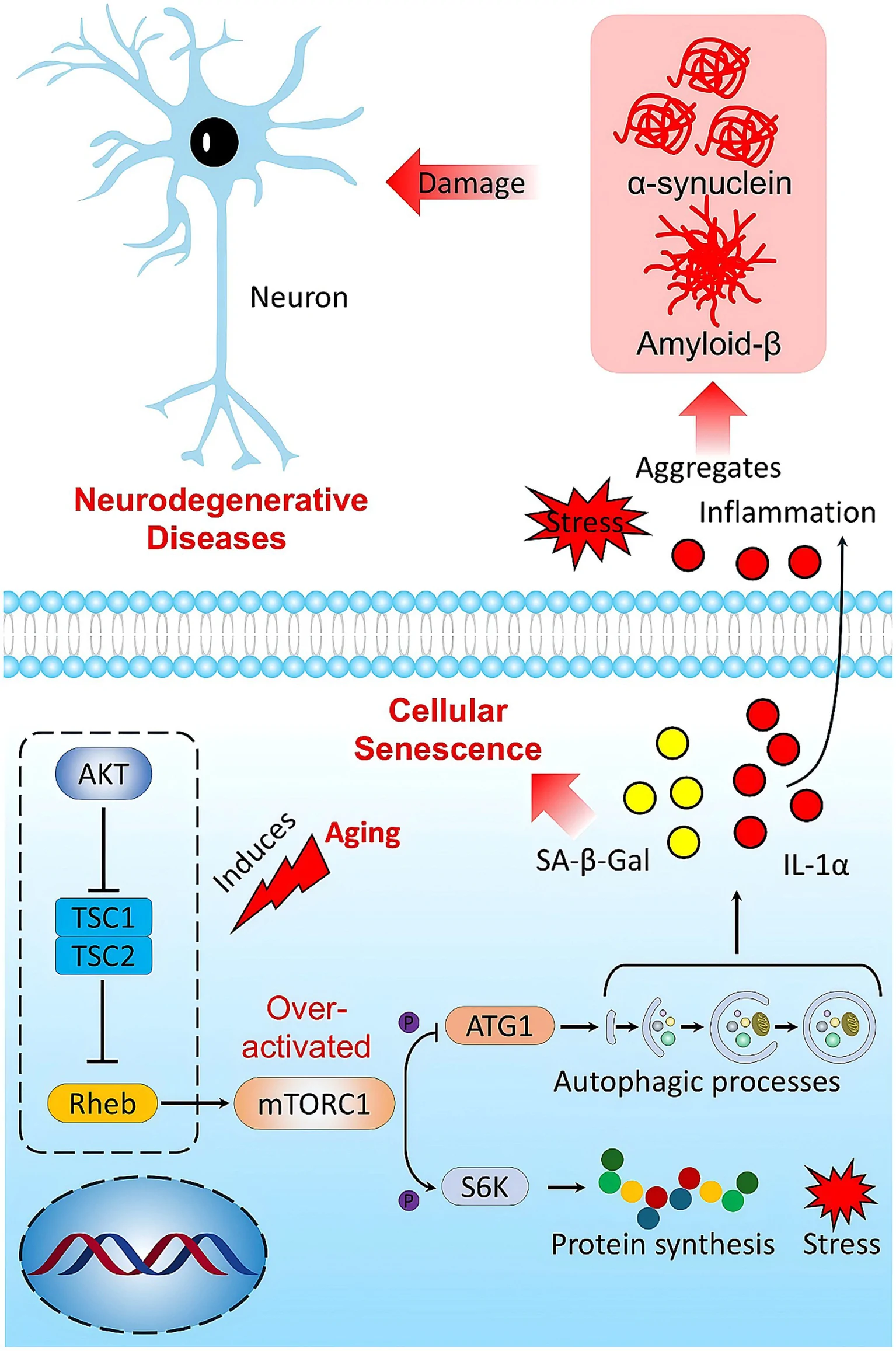
mTOR signaling pathway mediating cellular senescence in neurodegenerative diseases.

The mTOR pathway, comprising mammalian Target of Rapamycin Complex 1 (mTORC1) and mammalian Target of Rapamycin Complex 2 (mTORC2) complexes, serves as a central regulator of cellular senescence (46). As illustrated in Figure 1, this pathway integrates multiple upstream signals including Akt and p-S6K (47). Our comprehensive literature analysis reveals a biphasic effect of mTOR activation: while moderate mTORC1 activity promotes growth-related processes (48), sustained overactivation triggers cellular senescence through distinct mechanisms (49). In nutrient-rich environments, hyperactive mTORC1 drives excessive anabolic processes that ultimately increase endoplasmic reticulum stress (50), reactive oxygen species production (51), and DNA damage responses (52) - all hallmarks of accelerated senescence. This is evidenced by *in vitro* studies demonstrating that mTOR overactivation in human fibroblasts elevates SA-β-Gal activity and upregulates p16INK4A expression, two established biomarkers of cellular senescence (53).

In neurodegenerative diseases, the dysregulation of mTOR activity has profound implications. In AD, over-activation of mTOR contributes to the accumulation of tau tangles, reduced synaptic plasticity, and impaired autophagy (54, 55). Recent studies have shown that inhibiting mTOR can reduce tau phosphorylation and enhance memory in AD animal models, indicating its therapeutic potential (56, 57). In PD, mTOR signaling is involved in regulating the clearance of *α*-synuclein aggregates, and its dysregulation accelerates neurodegeneration (58, 59). Modulating mTOR activity holds promise for enhancing autophagy, restoring protein homeostasis, and reducing the accumulation of toxic protein aggregates, which are characteristic features of neurodegenerative diseases (60, 61).

The mTOR pathway also plays a prominent role in the aging - related aspects of neurodegenerative diseases. For example, in AD, hyperactivated mTOR has been observed in both the brain and peripheral lymphocytes, and this aberration is associated with the cognitive decline that occurs with aging in AD patients (28). As a key factor in the aging process, mTOR activation promotes anabolic processes while suppressing autophagy. This imbalance leads to the accumulation of damaged proteins and organelles, such as Aβ and tau tangles, which are hallmarks of AD. Interestingly, rapamycin - mediated inhibition of mTOR has been shown to reduce Aβ levels and improve cognitive function in AD mouse models (62). Moreover, mTOR activation in microglial cells can increase Trem2 expression, potentially enhancing Aβ clearance and halting the progression of AD pathology (63).

However, the role of mTOR in neurodegeneration is complex, as it is also involved in immune - mediated clearance. Dual PI3K/mTOR inhibitors like NVP - BEZ235 have shown potential in inhibiting tumor growth and may also provide protection against neurodegeneration by reducing senescence (64, 65). p-mTOR inhibitors are emerging as effective gerosuppressors, capable of delaying senescence and improving cellular health, with promising potential applications in managing neurodegenerative diseases (66, 67).

#### 2.2.2 AMPK signaling pathway

**Figure 2:**
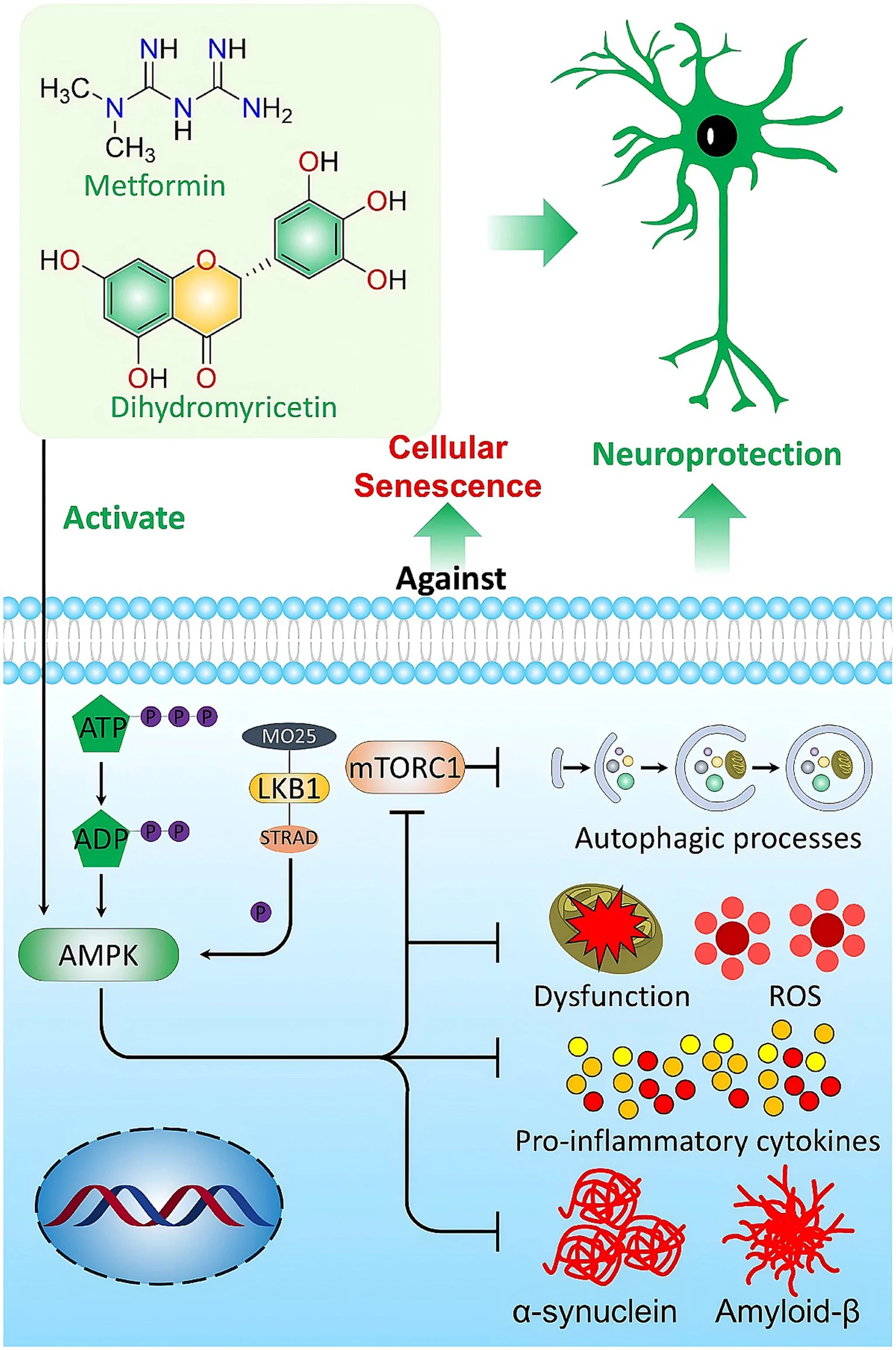
AMPK signaling pathway mediating cellular senescence in neurodegenerative diseases.

Adenosine 5′-monophosphate (AMP)-activated protein kinase (AMPK) is a crucial regulator of cellular energy homeostasis and metabolic stress. As Figure 2, the AMPK pathway is intricately involved in numerous cellular processes relevant to aging and neurodegeneration.

The activation of AMPK initiates a series of beneficial cellular responses. It promotes mitochondrial biogenesis, leading to the creation of new mitochondria for enhanced energy production. It also triggers autophagy, a process that clears damaged proteins, protecting cells from the accumulation of cellular waste. These processes collectively serve as a defense against cellular senescence, ensuring cell vitality and longevity (68, 69).

In neurodegenerative diseases, oxidative stress and mitochondrial dysfunction are major contributors to aging and neurodegeneration. AMPK activation has been shown to mitigate these threats (68, 69). In AD, AMPK activation has dual beneficial effects. It reduces amyloid-*β*-induced neurotoxicity and inhibits the accumulation of tau aggregates (70, 71). In AD models, AMPK activation rejuvenates mitochondrial function and reduces neuroinflammation, thus attenuating cognitive decline in preclinical models of aging (72). Metformin, an AMPK activator, modulates the AMPK/mTOR axis to enhance memory and prevent Aβ plaque deposition, combating aging - related neurodegeneration (73, 74). In PD, AMPK acts as a protector. It maintains mitochondrial function and safeguards dopaminergic neurons from *α*-synuclein - induced toxicity (75, 76). In HD, AMPK activation reduces protein aggregation, improving neuronal survival (77, 78). AMPK functions as a critical energy sensor. Upon detection of low cellular energy, it activates processes such as mitochondrial biogenesis, lipid metabolism, and autophagy. In neurodegenerative diseases, AMPK activation combats neuronal apoptosis, oxidative stress, and mitochondrial dysfunction, all of which are worsened by aging. Compounds like dihydromyricetin activate the AMPK/SIRT1 signaling pathway, defending against AD in rat models (79). FGF21, through AMPK - dependent mechanisms, suppresses cerebrovascular aging, enhances mitochondrial biogenesis, and restrains the p53 signaling pathway, which often goes awry in aging brains (80).

Overall, AMPK activation is a highly promising strategy in the battle against neurodegenerative diseases as it targets mechanisms closely associated with brain aging in preclinical models; whether these benefits translate to patients remains to be determined.

#### 2.2.3 Nrf2 signaling pathway

**Figure 3:**
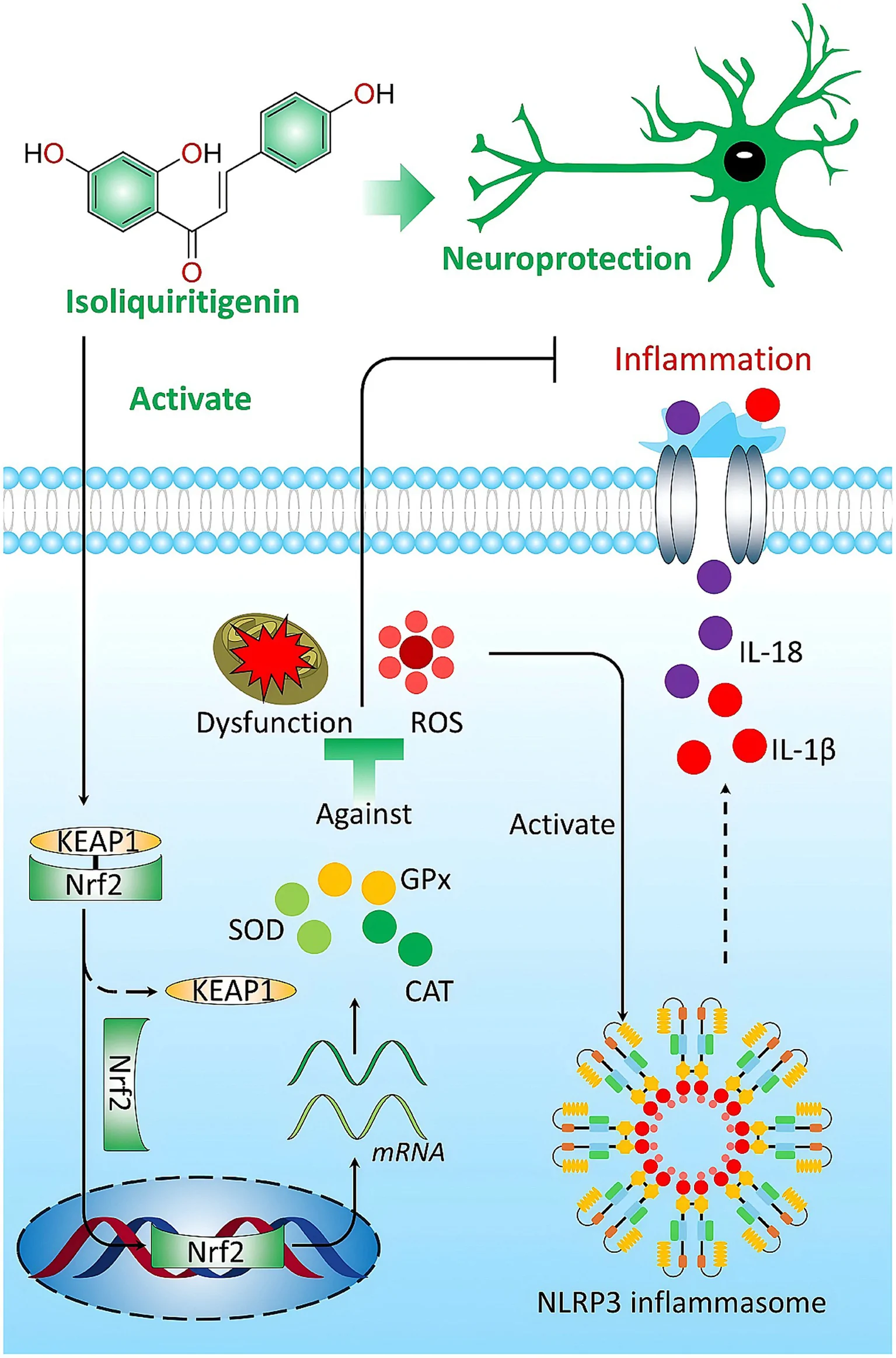
Nrf2 signaling pathway mediating cellular senescence in neurodegenerative diseases.

The Nrf2 pathway constitutes a critical regulatory axis in the intersection of aging and neurodegenerative diseases (Figure 3). Under basal conditions, Nrf2 is sequestered in the cytoplasm by Keap1 and maintained at low levels. Upon exposure to oxidative stress or electrophilic insults, Nrf2 dissociates from Keap1 and translocates to the nucleus, where it binds to antioxidant response elements (AREs) to initiate transcription of genes encoding antioxidant and detoxifying enzymes, including superoxide dismutase (SOD), catalase (CAT), and glutathione peroxidase (GPx) (81–84). This transcriptional program enhances cellular antioxidant capacity and mitigates free radical-mediated damage, thereby attenuating cellular senescence.

In Parkinson’s disease, Nrf2 activation in dopaminergic neurons attenuates mitochondrial dysfunction by reducing oxidative stress and enhancing mitochondrial biogenesis, thereby mitigating neuronal loss and slowing disease progression (85–87). Furthermore, Nrf2 modulates neuroinflammation by suppressing NLRP3 inflammasome activation (88, 89). In Alzheimer’s disease, Nrf2 activation protects neurons by reducing oxidative damage, maintaining mitochondrial function, and promoting synaptic plasticity (90–92). Natural compounds that activate Nrf2, such as *Centella asiatica* and isoliquiritigenin, have demonstrated neuroprotective effects in aging and neurodegenerative disease models (93, 94). Additionally, Nrf2 activation restores mitochondrial function and improves cognitive function in aged neurons, highlighting its therapeutic potential in age-related neurodegenerative disorders (95, 96).

#### 2.2.4 Sirtuin signaling pathway

**Figure 4:**
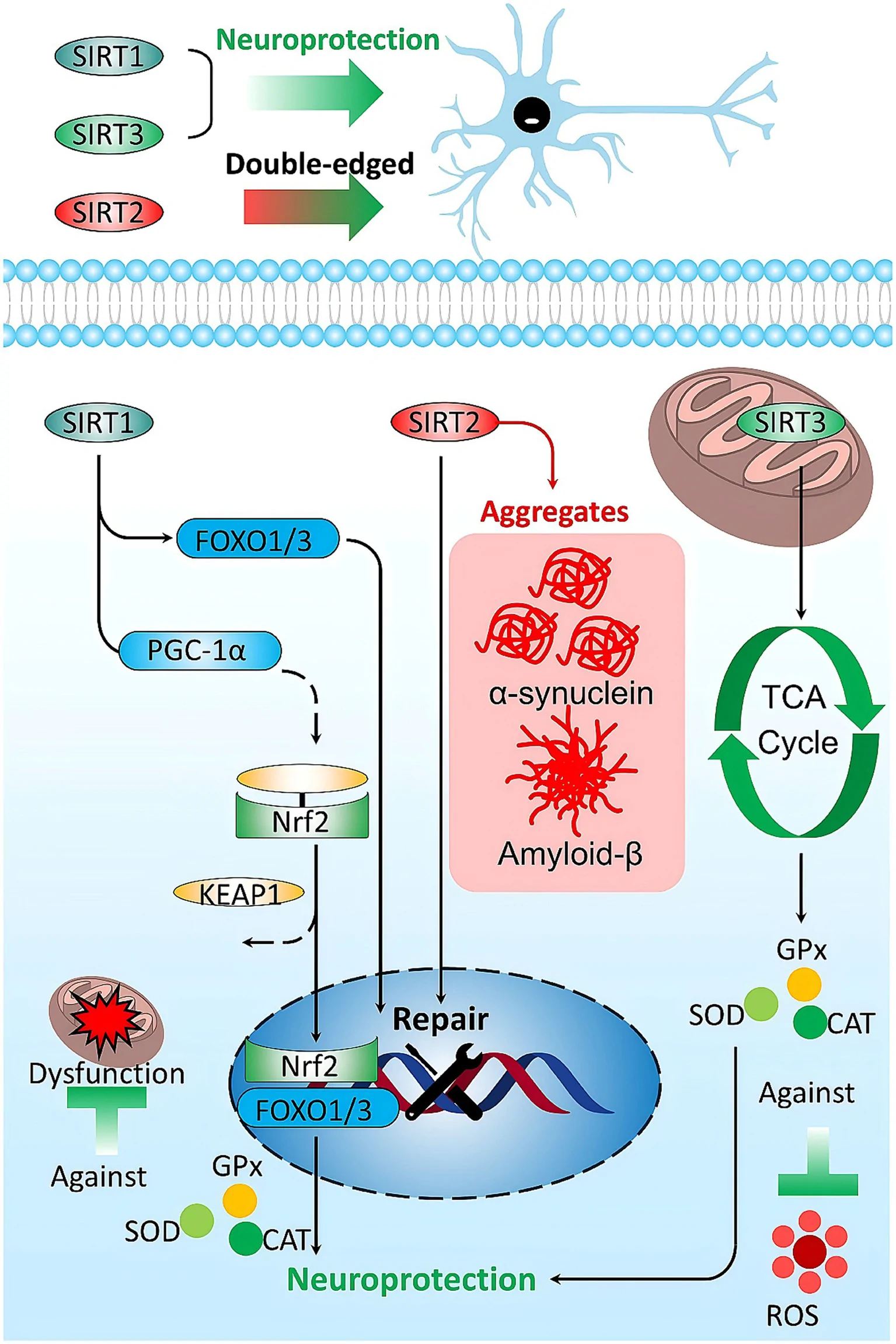
Sirtuin signaling pathway mediating cellular senescence in neurodegenerative diseases.

Silent information regulator proteins (Sirtuins), a family of NAD^+^ − dependent deacetylases, are at the heart of the complex web involving neurodegenerative diseases and aging processes. As showcased in Figure 4, the Sirtuins pathway is intricately linked to multiple cellular activities pertinent to these health challenges. In AD, SIRT1 activation enhances mitochondrial function, promotes clearance of misfolded proteins such as Aβ and tau, and ameliorates cognitive decline associated with aging (97–99). SIRT1 also interacts synergistically with the Nrf2 antioxidant pathway to promote mitochondrial biogenesis and attenuate oxidative stress, thereby counteracting the aging process. In contrast, SIRT2 is a double-edged sword in neurodegeneration. While it plays a part in promoting DNA repair and shoring up genomic stability, its overzealous activation in PD models has been known to fan the flames of *α*-synuclein toxicity by deacetylating key proteins implicated in protein aggregation, exacerbating the aging-related neurodegeneration (100, 101). Inhibiting SIRT2 activity has been floated as a possible treatment strategy for PD, a bid to rein in the aging-induced neurodegeneration (102). SIRT3, localized within mitochondria, plays a critical role in mitigating mitochondrial dysfunction and oxidative stress in neurodegenerative diseases. SIRT3 activation can supercharge mitochondrial bioenergetics and tamp down oxidative stress in PD and AD models, shielding cells from damage and enhancing survival, a crucial defense against aging-related neurodegeneration (103, 104). The harmonious interaction between SIRT3 and Nrf2 further accentuates the significance of mitochondrial function in neurodegeneration and the latent therapeutic benefits of targeting sirtuin signaling to combat aging-induced neurodegeneration (105).

## 3 Therapeutic potential of TCM active components in neurodegenerative diseases through cellular senescence modulation

The compounds reviewed herein were selected based on three criteria: (i) documented ethnopharmacological use in TCM or traditional Asian medical systems, or established integration into contemporary TCM research frameworks; (ii) demonstrated mechanistic activity in modulating cellular senescence pathways (e.g., SASP, mTOR, AMPK, Nrf2, sirtuins) in the context of NDDs; and (iii) availability of preclinical mechanistic studies enabling molecular-level analysis (7, 8). While ginsenosides, ginkgo biloba extract, tanshinone IIA, *Lycium barbarum* polysaccharide, and huperzine A are unequivocally derived from TCM botanicals (*Panax ginseng*, *Ginkgo biloba*, *Salvia miltiorrhiza*, *Lycium barbarum*, and *Huperzia serrata*, respectively), curcumin (from *Curcuma longa*) was included due to its extensive study within TCM-inspired anti-aging and neuroprotection research (11, 57). This selection spans diverse chemical classes—saponins, flavonoids, phenanthraquinones, polysaccharides, alkaloids, and polyphenols—to enable a comparative analysis of structurally distinct senescence modulators.

TCM offers a rich reservoir of bioactive compounds with antioxidative, anti-inflammatory, and senescence-modulating properties. This review explores the therapeutic potential of key TCM components, including ginkgo biloba extract, Tanshinone IIA, *Lycium barbarum* polysaccharide (LBP), Huperzine A, curcumin, quercetin, and resveratrol, in modulating cellular senescence and combating neurodegeneration (Table 1).

**Table 1:** Mechanisms by which active compounds in Traditional Chinese Medicine regulate cellular senescence signaling pathways.

Names of active components in TCM	Senescent targets	Category	Refs
Ginsenoside	SIRT1/AMPK	Saponins	107, 108
Ginkgo biloba extract	AKT/eNOS	Flavonoids	120
Tanshinone IIA	A20-NF-κB-NLRP3	Phenanthraquinones	134
*Lycium barbarum* polysaccharide	AKT	Polysaccharides	146
Huperzine A	NF-kB	Alkaloids	151
Curcumin	MAPK/NF-κB	Polyphenols	158
Quercetin	PI3K/AKT	Flavonoids	175
Resveratrol	Akt/mTORC1/S6K1	Polyphenols	183

### 3.1 Ginsenoside

Ginsenosides, the bioactive components of ginseng, have demonstrated significant potential in regulating cellular senescence, a key driver of neurodegenerative diseases. Specifically, these compounds contribute to mitochondrial homeostasis by stimulating mitochondrial biogenesis and reducing oxidative stress, thereby protecting neurons from age-related damage (106). In addition, ginsenosides regulate critical signaling pathways, such as the p53/p21 cascade, which controls the onset of senescence, while also enhancing autophagy. This dual action enables the clearance of damaged proteins and organelles, thus delaying the progression of neurodegeneration (11, 12). Moreover, ginsenosides alleviate neuroinflammation by inhibiting microglial activation and suppressing cytokine release. At the same time, they activate the SIRT1/AMPK pathway, which plays a pivotal role in promoting cellular longevity and resilience (107, 108).

Specifically, Ginsenoside Rg1 has been shown to promote neurite outgrowth in hippocampal neurons. This enhancement of neural plasticity, combined with its ability to reduce neuronal damage, underscores its neuroprotective potential against neurodegeneration (109). Similarly, Ginsenoside Rb1 supports hippocampal neurons by reducing oxidative stress and promoting neurite growth, offering further protection against neurodegenerative processes (110). Furthermore, Ginsenoside Rg3 plays a critical role in preventing oxidative stress-induced astrocytic senescence. By ameliorating the senescence-associated paracrine effects in glial cells, it helps mitigate neuroinflammation and its downstream impact on neurodegeneration (111).

In addition, Ginsenoside F1 has been shown to delay cellular senescence through its ability to preserve telomere length, contributing to cellular longevity and the maintenance of neuronal integrity (112). Meanwhile, Ginsenoside Rb2 enhances autophagic processes in human dermal fibroblasts, improving cellular quality control by clearing damaged proteins and organelles. This mechanism is critical for preventing cellular aging and neurodegeneration (113). Similarly, Ginsenoside Rh2 has demonstrated the ability to reduce cellular senescence by mitigating harmful bystander effects, highlighting its broader potential in therapies targeting neurodegenerative diseases (114).

Finally, Ginsenoside Rd. and Ginsenoside Re have shown remarkable neuroprotective effects in Parkinson’s disease models. By protecting against neurotoxic insults and preserving mitochondrial function, these compounds further emphasize the neuroprotective roles of ginsenosides in aging and related diseases (115, 116). Taken together, these findings highlight the comprehensive therapeutic potential of ginsenosides in managing cellular senescence and neurodegeneration, offering a promising natural approach to the treatment of neurodegenerative diseases.

Ginsenosides exhibit relatively favorable safety profiles in traditional use, but their pharmacokinetic limitations warrant attention. Oral bioavailability of major ginsenosides (e.g., Rb1, Rg1) is low (<5%) due to poor membrane permeability and extensive intestinal bacterial metabolism (deglycosylation to aglycones such as compound K and 20 (S)-protopanaxadiol). These metabolites may possess distinct pharmacological activities from their parent compounds. Furthermore, ginsenosides can inhibit CYP3A4 and CYP2D6, raising potential drug–drug interaction concerns in elderly patients receiving polypharmacy (117).

### 3.2 Ginkgo biloba extract

Ginkgo biloba extract (GBE), a widely studied herbal remedy, demonstrates significant potential in regulating cellular senescence, a critical factor in neurodegenerative diseases. Its neuroprotective effects are primarily mediated through antioxidant, anti-inflammatory, and anti-apoptotic mechanisms. For example, GBE prevents oxidative stress-induced apoptosis in neuroblastoma cells by inhibiting p53 activation, a key pathway involved in cellular senescence and neurodegeneration (118). Additionally, GBE activates the redoxosome-p66Shc pathway to reduce oxidative glutamate toxicity in SH-SY5Y cells, thereby promoting neuronal survival (119). Bilobalide, a major bioactive component of GBE, further supports neurovascular health by inhibiting autophagy and enhancing angiogenesis via the Akt/eNOS pathway following ischemia–reperfusion injury (120).

GBE also exerts significant systemic effects on oxidative stress and inflammation, which are central contributors to aging and neurodegeneration. The flavonoid glycoside fraction of GBE alleviates antioxidant imbalances, protecting against vanadium-induced brain damage (121). Similarly, ginkgetin, another active component of GBE, targets the STING pathway to mitigate inflammation and cellular senescence, offering additional neuroprotection (122). Its systemic antioxidant properties are further supported by studies in liver ischemia–reperfusion injury models, where GBE effectively reduces oxidative damage and inflammatory markers (123, 124).

In AD, GBE exhibits cognitive-enhancing effects by inhibiting Aβ aggregation and improving synaptic plasticity. In 5 × FAD mouse models, GBE has been shown to reduce Aβ pathology, enhance neurogenesis, and rescue memory deficits caused by synaptic damage, highlighting its therapeutic potential in AD (125, 126). Moreover, GBE plays an important role in maintaining mitochondrial homeostasis and proteostasis. By restoring antioxidant balance and reducing tau fibril formation, GBE mitigates neurodegeneration in tauopathy models. Its ability to modulate autophagy further promotes the clearance of damaged proteins, delaying cellular senescence (127).

Beyond the central nervous system, GBE demonstrates therapeutic effects in aging-related systemic conditions. For instance, it exhibits hepatoprotective properties in methotrexate-induced hepatotoxicity and anti-aging effects in cardiac models of D-galactose-induced heart aging (128). GBE also regulates NMDA receptor subunit expression, which is essential for neuroprotection and synaptic function, further solidifying its role in combating age-related cognitive decline (129).

GBE has undergone large-scale clinical evaluation, most notably the GEM study (10), which demonstrated no significant benefit in preventing dementia in elderly individuals with normal cognition or mild cognitive impairment. However, subgroup analyses suggested potential efficacy in patients with neuropsychiatric symptoms. GBE exhibits good BBB penetration for ginkgolides and bilobalide, but its flavonoid constituents undergo extensive conjugation. Notably, GBE inhibits CYP3A4, CYP2C9, and platelet-activating factor, increasing bleeding risk when co-administered with anticoagulants—a critical consideration in elderly populations (117).

In summary, these findings highlight GBE’s multifaceted roles in regulating cellular senescence and underscore its broad therapeutic potential for neurodegenerative diseases and other aging-related conditions.

### 3.3 Tanshinone IIA

Tanshinone IIA has been shown to modulate oxidative stress, a primary contributor to cellular senescence and neurodegeneration. By regulating oxidative stress pathways, it effectively reduces ROS levels and protects against oxidative damage in various cellular and animal models. For instance, in liver aging models, Tanshinone IIA delays the aging process by modulating oxidative stress, providing evidence of its potential to protect other tissues, including the brain, from age-related damage (130, 131). In neuronal models, it prevents glutamate-induced oxidative toxicity by inhibiting mitochondrial dysfunction and MAPK activation, two critical pathways involved in cellular survival and senescence (132). These findings establish Tanshinone IIA as a promising neuroprotective agent capable of mitigating oxidative damage in neurodegenerative diseases.

In addition to its antioxidative effects, Tanshinone IIA regulates key signaling pathways associated with cellular senescence. For example, it activates the SIRT1/eNOS pathway, which plays a pivotal role in reducing endothelial senescence and improving vascular health. The activation of SIRT1, a major longevity regulator, is linked to anti-aging effects and enhanced cellular resilience, indicating that Tanshinone IIA may address vascular contributions to neurodegenerative diseases such as AD and PD (133). Furthermore, Tanshinone IIA modulates the A20-NFκB-NLRP3 inflammasome-catalase pathway, reducing inflammation and cellular senescence in vascular tissues, particularly in diabetic models, suggesting its broader therapeutic potential for neurodegenerative diseases (134, 135).

The neuroprotective effects of Tanshinone IIA are further supported by its influence on mitochondrial function, a key determinant of aging and neurodegenerative processes. Mitochondrial dysfunction, a hallmark of neurodegeneration, contributes to neuronal loss and cognitive decline. Tanshinone IIA enhances mitochondrial function and promotes mitochondrial biogenesis through activation of the Nrf2-mediated antioxidant defense system, which plays a critical role in mitigating oxidative damage and inflammation (136, 137). By improving mitochondrial health, Tanshinone IIA reduces cellular dysfunction, which is particularly relevant in neurodegenerative diseases where mitochondrial deficits exacerbate neuronal degeneration.

Moreover, Tanshinone IIA exerts significant anti-inflammatory effects, an essential component of combating cellular senescence and neurodegeneration. By modulating the NF-κB pathway, it suppresses the production of pro-inflammatory cytokines, which are elevated in senescent cells and contribute to tissue damage in the brain. In diabetic mouse models, Tanshinone IIA alleviates vascular senescence and inflammation by targeting the A20-NFκB-NLRP3 inflammasome-catalase pathway, further supporting its therapeutic potential in addressing age-related neurodegenerative diseases (138).

Recent advancements in nanotechnology offer new opportunities to enhance the therapeutic efficacy of Tanshinone IIA by improving its bioavailability and brain-targeted delivery. Nanoparticles loaded with Tanshinone IIA improve its stability, facilitate crossing of the blood–brain barrier (BBB), and enable precise targeting of senescent cells in the brain. Drug delivery systems, such as liposomal and hybrid nanoparticles, are emerging as critical tools for enhancing the neuroprotective effects of Tanshinone IIA and translating its therapeutic potential into clinical practice (139, 140).

Despite these promising developments, challenges remain in the clinical application of Tanshinone IIA. Its limited ability to cross the BBB presents a significant obstacle, necessitating further optimization of drug delivery systems to ensure effective brain targeting (141, 142). Additionally, the long-term safety and potential toxicity of Tanshinone IIA, particularly concerning nanoparticle accumulation in non-target organs, require thorough evaluation (143). The scalability and cost-effectiveness of nanoparticle-based delivery systems for widespread clinical use also remain critical areas for further research.

### 3.4 *Lycium barbarum* polysaccharide (LBP)

LBPs are widely recognized for their potent antioxidant properties, which play a critical role in mitigating oxidative stress—a major contributor to cellular aging and senescence. Studies have demonstrated that LBPs effectively reduce ROS accumulation in cells, thereby protecting against oxidative damage and delaying the onset of cellular senescence (144, 145). In primary hippocampal neurons, LBPs preserve mitochondrial function by activating the Akt signaling pathway, a crucial regulator of cell survival and neurogenesis (146).

In addition to their antioxidative effects, LBPs regulate key pathways involved in cellular senescence, such as the p53 pathway. *In vitro* studies have shown that LBPs inhibit p53 activation, thereby reducing cell cycle arrest and apoptosis in aging cells (147). Furthermore, LBPs protect human lens epithelial cells from oxidative stress-induced senescence, highlighting their potential application in age-related ocular diseases (148). In animal models of aging, LBPs alleviate cognitive decline, an effect attributed to their ability to reduce mitochondrial dysfunction and enhance cellular repair mechanisms (149).

LBPs are high-molecular-weight polysaccharides with poor intestinal absorption; their bioactive metabolites likely result from gut microbiota fermentation. While generally regarded as safe, large-scale clinical trials evaluating LBPs for neurodegenerative diseases are lacking. Their effects on systemic immunity and potential interactions with hypoglycemic agents require further investigation.

### 3.5 Huperzine A

Cellular senescence, characterized by irreversible cell cycle arrest, the SASP, and increased oxidative stress, is a critical factor in brain aging. Huperzine A has demonstrated the ability to mitigate these senescence-associated changes in neuronal cells. In AD and PD models, Huperzine A improves cognitive function and reduces neuroinflammation by suppressing oxidative stress and inflammatory pathways such as NF-κB (150–152). Furthermore, Huperzine A exhibits significant antioxidant effects by decreasing ROS production, which is often elevated in neurodegenerative diseases (153).

Huperzine A exerts its effects primarily through the PI3K/Akt signaling pathway, a key regulator of neuronal survival and cell cycle progression. Activation of this pathway by Huperzine A promotes neuronal survival and protects neurons from apoptosis and ferroptosis, a form of programmed cell death driven by oxidative stress and iron overload (154, 155). Additionally, Huperzine A enhances mitochondrial function, protecting neurons from oxidative damage and improving overall neuronal health (156). It also inhibits apoptosis by modulating apoptotic proteins such as Bax and Bcl-2, further preventing neurodegeneration (157).

Huperzine A possesses the strongest clinical pedigree among the compounds reviewed, having undergone Phase II–III trials in China for AD (9). It has been reported to penetrate the BBB in preclinical pharmacokinetic studies efficiently and has a favorable safety profile at standard doses (100–200 μg twice daily). However, its narrow therapeutic window and cholinergic side effects (nausea, dizziness, bradycardia) limit widespread use. As an acetylcholinesterase inhibitor with additional anti-senescence properties, it represents an ideal candidate for combination therapy with NMDA receptor modulators or mitochondrial protective agents.

### 3.6 Curcumin

Curcumin, a polyphenol derived from *Curcuma longa*, plays a significant role in mitigating cellular senescence, characterized by cell cycle arrest, SASP, and oxidative stress (158, 159). Curcumin addresses these factors through its antioxidant, anti-inflammatory, and epigenetic regulatory properties (160, 161). A key mechanism underlying its effects is the activation of sirtuin pathways, particularly SIRT1, which is essential for longevity and neuronal health. Curcumin elevates SIRT1 levels, improving mitochondrial function, reducing oxidative stress, and promoting neuronal survival (162). However, its effects on delaying senescence are influenced by cell type and environmental factors (163).

Curcumin also inhibits the MAPK/NF-κB signaling pathway, which is critical for inflammation and cellular senescence. In aged mice, curcumin suppresses NF-κB activation and reduces MAPK signaling, alleviating inflammation and protecting against cellular aging (164). This pathway modulation is particularly beneficial in neurodegenerative diseases, where chronic inflammation accelerates neuronal loss and cognitive decline (165). Moreover, curcumin enhances mitochondrial function by regulating CISD2, a protein crucial for mitochondrial homeostasis, improving cellular repair mechanisms and reducing age-related susceptibility to conditions like neurodegeneration and atrial fibrillation (166).

In models of Alzheimer’s disease, curcumin has shown the ability to prevent amyloid-beta (Aβ) aggregation and reduce tau pathology, both hallmarks of the disease. By reducing oxidative stress and inflammation, curcumin enhances synaptic plasticity and cognitive function (167). Preclinical formulation studies suggest that curcumin can be delivered across the blood–brain barrier, further supporting its therapeutic potential (168).

Beyond receptor-mediated signaling, curcumin and related polyphenols can directly interact with proteins through non-specific hydrophobic and hydrogen bonding, functioning as chemical chaperones or, at higher concentrations, protein denaturants (169, 170). In the context of Parkinson’s disease, curcumin has been shown to interact with *α*-synuclein monomers, promoting the formation of soluble, non-toxic oligomeric species that are off-pathway from fibrillar aggregation (171, 172). While this effectively sequesters α-synuclein into relatively inert aggregates, the long-term stability and potential toxicity of these curcumin-α-synuclein cross-linked species remain uncertain. Similar interactions have been reported with amyloid-β, where curcumin inhibits fibrillization but generates stable oligomeric intermediates. These findings suggest that the neuroprotective effects of curcumin in proteinopathies may partially derive from direct biophysical interference with protein aggregation rather than pure signaling pathway modulation. However, this mechanism is concentration-dependent: low micromolar concentrations (1–10 μM) favor specific signaling activation (Nrf2, SIRT1), whereas supraphysiological concentrations (>50–100 μM) risk non-specific protein cross-linking, membrane disruption, and metal ion chelation that could paradoxically impair cellular proteostasis (45, 170).

Despite promising preclinical data, curcumin faces formidable clinical translation barriers. Its poor oral bioavailability (<1% in humans), rapid systemic elimination (half-life ~1–2 h), and extensive hepatic conjugation limit achievable CNS concentrations (45). Furthermore, curcumin exhibits dose-dependent biphasic effects: while low concentrations (1–5 μM) activate Nrf2 and SIRT1, higher concentrations (>50 μM) can non-specifically denature proteins and chelate metal ions (Fe^2+^, Cu^2+^), potentially disrupting essential metalloenzyme function (169, 170). These properties necessitate advanced formulation strategies (nanoparticles, liposomes, phospholipid complexes) or structural analogs (e.g., C3-complex, Theracurmin) to improve bioavailability and achieve therapeutic CNS exposure.

### 3.7 Quercetin

Quercetin exhibits senolytic properties, effectively clearing senescent cells from tissues, including the brain. *In vitro* studies show that quercetin induces apoptosis in senescent pre-adipocytes and adipocytes, promoting tissue rejuvenation (173). Quercetin also inhibits endothelial cell senescence induced by oxidized LDL, a crucial mechanism for its neuroprotective effects, particularly since vascular dysfunction exacerbates neurodegeneration in AD and PD (174).

Quercetin regulates oxidative stress and promotes cell survival by activating the PI3K/Akt signaling pathway, which enhances resistance to apoptosis and supports tissue repair in neurodegeneration models (175). Additionally, quercetin modulates the AMPK pathway, which regulates cellular metabolism, further delaying senescence and maintaining cellular function, particularly in vascular smooth muscle cells (176). Its ability to reduce neuroinflammation and oxidative stress underscores its therapeutic potential for neurodegenerative diseases. Quercetin has been shown to reduce inflammation and protect neurons from age-related damage, ultimately improving cognitive function (177). Importantly, its ability to cross the blood–brain barrier enhances its effectiveness in treating neurodegenerative diseases (178).

As a broad-spectrum senolytic, quercetin’s lack of cell-type specificity poses theoretical risks for the CNS (37). Senescent cell clearance must be balanced against the preservation of neural stem cell niches and oligodendrocyte precursor populations that share senescence-associated markers (e.g., p16INK4a expression under stress) (20, 179). Chronic quercetin administration could potentially deplete these regenerative pools, impairing hippocampal neurogenesis and remyelination capacity in the aging brain. Moreover, quercetin inhibits topoisomerase II and may exert genotoxic effects at high concentrations (170), further underscoring the need for targeted delivery systems that restrict its action to pathological senescent cells.

Quercetin is the prototypical natural senolytic, most frequently studied in combination with dasatinib (D + Q) (180, 181). While D + Q has entered early-phase human trials for systemic senescence-related conditions (36, 181), its CNS applications remain unexplored. Quercetin exhibits moderate BBB penetration but undergoes rapid phase II metabolism. Its senolytic efficacy is concentration-dependent, and achieving therapeutic CNS concentrations without systemic toxicity represents a major formulation challenge.

### 3.8 Resveratrol

Resveratrol primarily exerts its anti-aging effects through the activation of sirtuins, particularly SIRT1, a key regulator of cellular stress response and senescence. While resveratrol typically promotes SIRT1 activation, it has also been shown to induce premature senescence under specific conditions by downregulating SIRT1 and SIRT2, indicating its effects may vary based on dosage and cellular environment (182).

Resveratrol modulates critical pathways involved in cellular senescence, including the Akt/mTORC1/S6K1 signaling axis, which regulates cell growth and survival. By inhibiting this pathway, resveratrol prevents excessive cell growth and reduces the accumulation of senescent cells, thereby mitigating aging and neurodegeneration (183). It also activates the AMPK-FOXO3 signaling cascade, which enhances cellular stress resistance and longevity (184).

Resveratrol’s neuroprotective effects are further supported by its role in improving mitochondrial health. By activating SIRT1 and AMPK pathways, resveratrol enhances mitochondrial biogenesis and function, reducing oxidative stress and promoting neuronal survival (96). In human airway epithelial cells, resveratrol reduces senescence induced by cigarette smoke extract by modulating the miR-34a/SIRT1/NF-κB pathway, suggesting its potential to mitigate oxidative damage in neurodegenerative diseases (185). These combined effects highlight resveratrol’s therapeutic potential in combating cellular senescence and promoting neuronal health.

Resveratrol exemplifies the hormetic principle in neuroprotection: low doses (5–20 μM) activate SIRT1 and AMPK, conferring anti-senescence and mitochondrial protective effects, whereas higher doses (>100 μM) can induce oxidative stress, DNA damage, and paradoxical cellular senescence through SIRT1 downregulation (182, 186). Resveratrol’s stilbene structure enables it to intercalate into protein aggregates and modulate their conformation; in PD models, it interacts with *α*-synuclein fibrils, potentially remodeling them into less toxic species (172). However, this direct protein-binding activity is non-selective and may interfere with normal protein–protein interactions essential for synaptic function. Additionally, resveratrol’s rapid metabolism to sulfate and glucuronide conjugates *in vivo* generates metabolites with unknown protein-binding profiles, further complicating the interpretation of its mechanism of action in clinical settings (186, 187).

Resveratrol’s clinical translation is severely hampered by its extremely low bioavailability (<1% of oral dose reaches systemic circulation as free resveratrol) and rapid first-pass metabolism (187). Human plasma concentrations after oral administration (500–1,000 mg) typically reach only 10–50 ng/mL, far below the micromolar concentrations used in most *in vitro* neuroprotection studies. Repeated high-dose administration may paradoxically induce cellular senescence through SIRT1 downregulation and DNA damage (182, 186). Novel delivery systems (lipid nanoparticles, prodrugs, sustained-release formulations) are essential to achieve therapeutic CNS exposure.

### 3.9 Comparative analysis of TCM-derived senescence modulators

The bioactive compounds reviewed above exhibit substantial overlap in their molecular targets, with most converging on the AMPK-mTOR-Nrf2-SIRT1 axis (Table 1). However, important distinctions emerge when considering target specificity, senescence-modulating modality, and disease relevance:

Ginsenosides (particularly Rg3 and Rh2) demonstrate relatively selective targeting of glial senescence and SASP attenuation, making them particularly relevant for AD and PD where astrocytic and microglial senescence drive neuroinflammation (111, 114). In contrast, quercetin acts as a broad-spectrum senolytic capable of clearing senescent cells across multiple lineages, but this lack of specificity raises concerns regarding off-target elimination of non-senescent or regenerating cell populations (e.g., neural stem cells, oligodendrocyte precursor cells) (20, 37, 179). Curcumin and resveratrol function primarily as senomorphics—suppressing SASP without necessarily eliminating senescent cells—through SIRT1-mediated epigenetic modulation (162, 182). Tanshinone IIA distinguishes itself through vascular-protective effects via the SIRT1/eNOS pathway, suggesting particular utility in mixed dementia or cerebrovascular contributions to NDDs (133).

When stratified by evidence level, huperzine A possesses the strongest clinical pedigree, having undergone Phase II–III trials for AD (9). Ginkgo biloba extract (EGb761) has been evaluated in large-scale clinical trials (e.g., GEM study) (10), though its effects on cellular senescence per se remain underexplored in human subjects. The remaining compounds—ginsenosides, tanshinone IIA, LBP, curcumin, quercetin, and resveratrol—are supported predominantly by *in vitro* and rodent studies, with human data limited to pharmacokinetic pilot studies or small biomarker trials.

A critical translational parameter is BBB permeability (188). Huperzine A and ginkgolides have been reported to cross the BBB in preclinical pharmacokinetic studies, explaining their clinical viability. Curcumin and resveratrol exhibit poor bioavailability and rapid hepatic glucuronidation, necessitating nanoparticle formulations or structural modifications to achieve CNS therapeutic concentrations (45, 187). Quercetin’s BBB penetration is moderate but concentration-dependent, while tanshinone IIA requires liposomal or nanoparticle delivery for effective CNS targeting (141, 142). These pharmacokinetic differences must inform compound selection for specific NDDs based on target brain region and required tissue exposure.

## 4 Challenges and new opportunities

Despite promising preclinical data, these TCM compounds face significant translational barriers. Polyphenols such as curcumin and resveratrol exhibit poor oral bioavailability (<1–2%) and extensive hepatic metabolism, with *in vitro* effective concentrations (1–50 μM) far exceeding achievable plasma levels (45, 187, 189). Many TCM compounds also modulate cytochrome P450 enzymes, raising drug–drug interaction concerns in elderly patients receiving polypharmacy (117). Furthermore, broad-spectrum senolytics like quercetin lack cell-type specificity, potentially depleting neural stem cells and oligodendrocyte precursor cells that share senescence markers (179, 190). Nanoparticle delivery systems, while improving BBB penetration, introduce immunogenicity, long-term biodistribution uncertainties, and regulatory hurdles (191, 192). Finally, natural senolytics may achieve enhanced efficacy through rational combination regimens—exemplified by dasatinib plus quercetin (D + Q), which has entered early-phase human trials (36, 180, 181, 193)—yet their CNS applications remain unexplored.

Nanoparticles have emerged as a promising strategy for delivering bioactive compounds commonly used in TCM, such as curcumin, resveratrol, and ginsenosides. These compounds are often characterized by poor solubility and bioavailability, which limits their therapeutic efficacy. However, encapsulation within nanomaterials has been shown to significantly enhance their pharmacokinetics and therapeutic potential. For example, resveratrol-loaded nanoparticles have demonstrated improved efficacy in reducing oxidative stress and modulating senescence pathways through the enhancement of SIRT1 activity, a key regulator of aging and cell survival (194, 195). Additionally, nanoparticles engineered to cross the blood–brain barrier or interact with specific cellular receptors enable targeted delivery of TCM compounds to senescent cells in the brain. This targeted delivery is particularly crucial for improving therapeutic outcomes in neurodegenerative diseases, where conventional drug delivery systems often fail to effectively reach the central nervous system (196).

**Figure 5:**
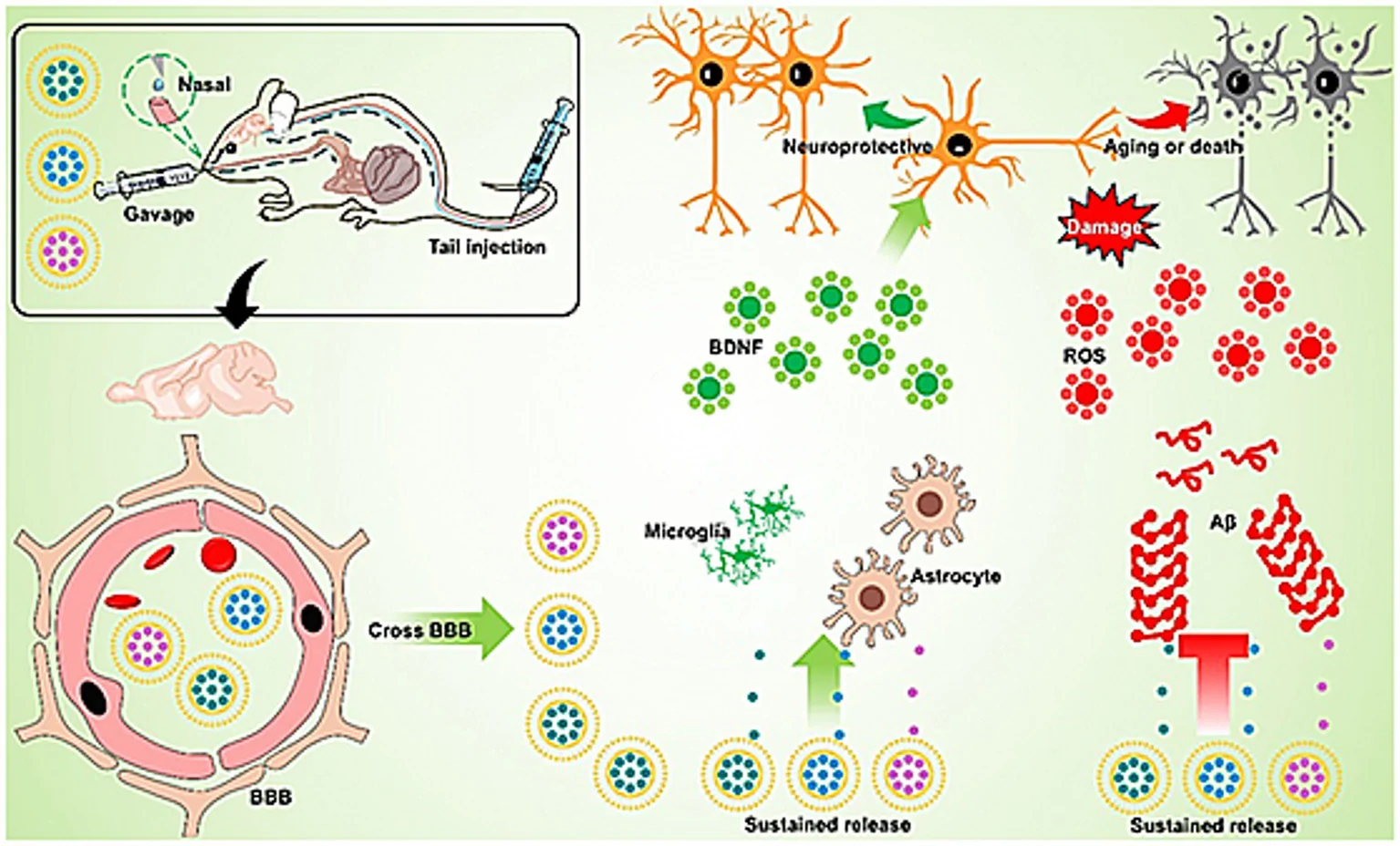
The therapeutic potential of nanoparticle-encapsulated TCM compounds in the treatment of neurodegenerative diseases. Nanoparticle-encapsulated TCM compounds (represented by the yellow-ringed spheres) can be administered through various drug delivery routes, such as nasal administration, oral gavage, or intravenous (tail) injection, cross the blood–brain barrier (BBB), and achieve sustained-release effects. Once in the brain, these nanoparticles act on senescent glial cells and neurons, enhancing neuroprotective signaling (e.g., BDNF) while suppressing cellular senescence, reactive oxygen species (ROS), and amyloid-β (Aβ) aggregation. Microglia and astrocytes are depicted proportionate to their relative sizes in the brain. By effectively targeting senescent cells and inhibiting cellular aging, these nanoparticle-based therapies hold significant potential for treating neurodegenerative diseases.

Moreover, combining nanomaterials with TCM compounds offers synergistic, multi-target therapeutic benefits. For instance, curcumin-loaded nanoparticles not only mitigate oxidative stress but also enhance autophagy and reduce inflammation—processes that play critical roles in cellular senescence and neurodegeneration (197, 198). In particular, nanoparticles carrying curcumin and other TCM compounds have shown significant promise in treating age-related diseases by modulating key cellular pathways, such as NF-κB and MAPK, both of which are closely associated with inflammation and cellular senescence (199). Furthermore, the ability of these nanoparticles to target specific senescent cells within the central nervous system enhances their neuroprotective effects, making them a highly attractive therapeutic option for neurodegenerative conditions (Figure 5).

Despite these advances, several challenges remain in the application of nanomaterials for TCM-based therapies in neurodegenerative diseases. One significant challenge is the limited understanding of the long-term safety and potential toxicity of nanoparticles, particularly their accumulation in organs such as the liver and kidneys. Optimizing the size, charge, and surface properties of nanoparticles is essential to minimize adverse effects and maximize their efficiency in delivering TCM compounds to target sites. Another critical hurdle is the scalability of producing nanomaterial-based TCM formulations for clinical applications, especially in terms of cost, consistency, and reproducibility.

## 5 Conclusion

The active components of TCM present significant potential for treating neurodegenerative diseases by targeting and modulating cellular senescence (200). Bioactive compounds such as ginsenosides, curcumin, and resveratrol have demonstrated promising effects in reducing cellular aging, protecting neurons, and alleviating symptoms associated with conditions like AD and PD. Despite these encouraging findings, challenges related to the bioavailability, pharmacokinetics, and clinical translation of these compounds remain significant barriers to their widespread use. Nevertheless, with ongoing research and advancements in technology—such as nanoparticle-based delivery systems—TCM-based therapies have the potential to become a valuable addition to existing treatment strategies for neurodegenerative diseases.

In particular, the low selectivity of natural senolytics may lead to the removal of regenerative cells that should be preserved, which could be dangerous for the brain. Furthermore, many polyphenols exhibit concentration-dependent biphasic effects and can directly interact with pathological proteins, forming cross-linked oligomers with uncertain long-term consequences.

## Data Availability

The original contributions presented in the study are included in the article/supplementary material, further inquiries can be directed to the corresponding author.

## Glossary

Aβ: Amyloid-β
AD: Alzheimer’s disease
AMPK: Adenosine 5′-monophosphate (AMP)-activated protein kinase
ARE: Antioxidant response element
CAT: Catalase
GBE: Ginkgo biloba extract
GPx: Glutathione peroxidase
HD: Huntington’s disease
LBP: *Lycium barbarum* polysaccharide
LDL: Low-Density Lipoprotein
mHTT: Mutant huntingtin
MMPs: Matrix metalloproteinases
mTOR: Mammalian target of rapamycin
mTORC1: Mammalian Target Of Rapamycin Complex 1
mTORC2: Mammalian Target Of Rapamycin Complex 2
NDDs: Neurodegenerative diseases
PD: Parkinson’s disease
ROS: Reactive oxygen species
SASP: Senescence-associated secretory phenotype
SOD: Superoxide dismutase
TCM: Traditional Chinese Medicine

## References

[1] Hou Y Dan X Babbar M Wei Y Hasselbalch SG Croteau DL . Ageing as a risk factor for neurodegenerative disease. Nat Rev Neurol. (2019) 15:565–81. doi: 10.1038/s41582-019-0244-7, 31501588

[2] Scheltens P De Strooper B Kivipelto M Holstege H Chételat G Teunissen CE . Alzheimer's disease. Lancet. (2021) 397:1577–90. doi: 10.1016/S0140-6736(20)32205-4, 33667416 PMC8354300

[3] Maphis N Xu G Kokiko-Cochran ON Jiang S Cardona A Ransohoff RM . Reactive microglia drive tau pathology and contribute to the spreading of pathological tau in the brain. Brain. (2015) 138:1738–55. doi: 10.1093/brain/awv081 25833819 PMC4542622

[4] Musi N Valentine JM Sickora KR Baeuerle E Thompson CS Shen Q . Tau protein aggregation is associated with cellular senescence in the brain. Aging Cell. (2018) 17:e12840. doi: 10.1111/acel.12840, 30126037 PMC6260915

[5] Gorgoulis V Adams PD Alimonti A Bennett DC Bischof O Bishop C . Cellular senescence: defining a path forward. Cell. (2019) 179:813–27. doi: 10.1016/j.cell.2019.10.005, 31675495

[6] Hernandez-Segura A Nehme J Demaria M. Hallmarks of cellular senescence. Trends Cell Biol. (2018) 28:436–53. doi: 10.1016/j.tcb.2018.02.001, 29477613

[7] Newman DJ Cragg GM. Natural products as sources of new drugs over the nearly four decades from 01/1981 to 09/2019. J Nat Prod. (2020) 83:770–803. doi: 10.1021/acs.jnatprod.9b01285, 32162523

[8] Atanasov AG Zotchev SB Dirsch VM Supuran CT. Natural products in drug discovery: advances and opportunities. Nat Rev Drug Discov. (2021) 20:200–16. doi: 10.1038/s41573-020-00114-z, 33510482 PMC7841765

[9] Ma X Tan C Wang H Feng S Wang R Tan L . Huperzine A: a novel treatment for Alzheimer's disease. Curr Med Chem. (2018) 25:2243–54.

[10] DeKosky ST Williamson JD Fitzpatrick AL Kronmal RA Ives DG Saxton JA . Ginkgo biloba for prevention of dementia: a randomized controlled trial. JAMA. (2008) 300:2253–62. doi: 10.1001/jama.2008.683 19017911 PMC2823569

[11] Du X Lou N Hu S Xiao R Chu C Huang Q . Anti-aging of the nervous system and related neurodegenerative diseases with Chinese herbal medicine. Am J Alzheimers Dis Other Dement. (2023) 38:15333175231205445. doi: 10.1177/15333175231205445, 37818604 PMC10624054

[12] Wang L Wang J Yang Z Wang Y Zhao T Luo W . Traditional herbs: mechanisms to combat cellular senescence. Aging. (2023) 15:14473–505. doi: 10.18632/aging.205269, 38054830 PMC10756111

[13] Chinta SJ Woods G Demaria M Rane A Zou Y McQuade A . Cellular senescence is induced by the environmental neurotoxin Paraquat and contributes to neuropathology linked to Parkinson's disease. Cell Rep. (2018) 22:930–40. doi: 10.1016/j.celrep.2017.12.092, 29386135 PMC5806534

[14] Dehay B Martinez-Vicente M Caldwell GA Caldwell KA Yue Z Cookson MR . Lysosomal impairment in Parkinson's disease. Mov Disord. (2013) 28:725–32. doi: 10.1002/mds.25462, 23580333 PMC5131721

[15] Crotti A Benner C Kerman BE Gosselin D Lagier-Tourenne C Zuccato C . Mutant huntingtin promotes autonomous microglia activation via myeloid lineage-determining factors. Nat Neurosci. (2014) 17:513–21. doi: 10.1038/nn.3668, 24584051 PMC4113004

[16] Glajch KE Sadri-Vakili G. Epigenetic mechanisms involved in Huntington's disease pathogenesis. J Huntingtons Dis. (2015) 4:1–15. doi: 10.3233/jhd-159001 25813218

[17] Baker DJ Childs BG Durik M Wijers ME Sieben CJ Zhong J . Naturally occurring P16 (Ink4a)-positive cells shorten healthy lifespan. Nature. (2016) 530:184–9. doi: 10.1038/nature16932, 26840489 PMC4845101

[18] Salminen A Kauppinen A Kaarniranta K. Emerging role of Nf-Κb signaling in the induction of senescence-associated secretory phenotype (Sasp). Cell Signal. (2012) 24:835–45. doi: 10.1016/j.cellsig.2011.12.006, 22182507

[19] Bussian TJ Aziz A Meyer CF Swenson BL van Deursen JM Baker DJ. Clearance of senescent glial cells prevents tau-dependent pathology and cognitive decline. Nature. (2018) 562:578–82. doi: 10.1038/s41586-018-0543-y, 30232451 PMC6206507

[20] Zhang P Kishimoto Y Grammatikakis I Gottimukkala K Cutler RG Zhang S . Senolytic therapy alleviates aβ-associated oligodendrocyte progenitor cell senescence and cognitive deficits in an Alzheimer's disease model. Nat Neurosci. (2019) 22:719–28. doi: 10.1038/s41593-019-0372-9, 30936558 PMC6605052

[21] Bhat R Crowe EP Bitto A Moh M Katsetos CD Garcia FU . Astrocyte senescence as a component of Alzheimer's disease. PLoS One. (2012) 7:e45069. doi: 10.1371/journal.pone.0045069, 22984612 PMC3440417

[22] Wang Y Wang Z Guo S Li Q Kong Y Sui A . Svhrsp alleviates age-related cognitive deficiency by reducing oxidative stress and Neuroinflammation. *Antioxidants (Basel)*. (2024) 13:628. doi: 10.3390/antiox13060628, 38929067 PMC11200511

[23] Hong H Kim BS Im HI. Pathophysiological role of Neuroinflammation in neurodegenerative diseases and psychiatric disorders. Int Neurourol J. (2016) 20:S2–7. doi: 10.5213/inj.1632604.302, 27230456 PMC4895907

[24] Heneka MT Carson MJ El Khoury J Khoury Joseph El Landreth Gary E Brosseron Frederic . Lancet Neurol (2015) 14 388–405 doi: 10.1016/S1474-4422(15)70016-5 25792098 PMC5909703

[25] Martín-Montañez E Millon C Boraldi F Garcia-Guirado F Pedraza C Lara E . Igf-ii promotes neuroprotection and neuroplasticity recovery in a Long-lasting model of oxidative damage induced by glucocorticoids. Redox Biol. (2017) 13:69–81. doi: 10.1016/j.redox.2017.05.012, 28575743 PMC5454142

[26] Ramos V Reis M Ferreira L Silva AM Ferraz R Vieira M . Stalling the course of neurodegenerative diseases: could Cyanobacteria constitute a new approach toward therapy? Biomolecules. (2023) 13:1444. doi: 10.3390/biom13101444, 37892126 PMC10604708

[27] Ovadya Y Landsberger T Leins H Vadai E Gal H Biran A. Impaired immune surveillance accelerates accumulation of senescent cells. Nat Commun. (2018) 9:5435. 30575733 10.1038/s41467-018-07825-3 PMC6303397

[28] Paccalin M Pain-Barc S Pluchon C Paul C Besson MN Carret-Rebillat AS . Activated Mtor and Pkr kinases in lymphocytes correlate with memory and cognitive decline in Alzheimer's disease. Dement Geriatr Cogn Disord. (2006) 22:320–6. doi: 10.1159/000095562, 16954686

[29] Kam TI Hinkle JT Dawson TM Dawson VL. Microglia and astrocyte dysfunction in Parkinson's disease. Neurobiol Dis. (2020) 144:105028. doi: 10.1016/j.nbd.2020.105028, 32736085 PMC7484088

[30] Wang QQ Yin G Huang JR Xi SJ Qian F Lee RX . Ionizing radiation-induced brain cell aging and the potential underlying molecular mechanisms. Cells. (2021) 10:3570. doi: 10.3390/cells10123570, 34944078 PMC8700624

[31] Vekrellis K Xilouri M Emmanouilidou E Rideout HJ Stefanis L. Pathological roles of α-synuclein in neurological disorders. Lancet Neurol. (2011) 10:1015–25. 22014436 10.1016/S1474-4422(11)70213-7

[32] Gao HM Liu B Zhang W Hong JS. Critical role of microglial NADPH oxidase-derived free radicals in the in vitro MPTP model of Parkinson’s disease. FASEB J. (2003) 17:1954–6. doi: 10.1096/fj.03-0109fje 12897068

[33] Vicente Miranda H Szegö ÉM Oliveira LMA Breda C Darendelioglu E de Oliveira RM . Glycation potentiates α-synuclein-associated neurodegeneration in synucleinopathies. Brain. (2017) 140:1399–419. doi: 10.1093/brain/awx056, 28398476

[34] Richarme G Mihoub M Dairou J Bui LC Leger T Lamouri A. Parkinsonism-associated protein DJ-1/Park7 is a major protein deglycase that repairs methylglyoxal- and glyoxal-glycated cysteine, arginine, and lysine residues. J Biol Chem. (2015) 290:1885–97. doi: 10.1074/jbc.M114.597815, 25416785 PMC4340429

[35] van Deursen JM. The role of senescent cells in ageing. Nature. (2014) 509:439–46. doi: 10.1038/nature13193, 24848057 PMC4214092

[36] Justice JN Nambiar AM Tchkonia T LeBrasseur NK Pascual R Hashmi SK . Senolytics in idiopathic pulmonary fibrosis: results from a first-in-human, open-label, pilot study. EBioMedicine. (2019) 40:554–63. doi: 10.1016/j.ebiom.2018.12.052, 30616998 PMC6412088

[37] Kirkland JL Tchkonia T. Senolytic drugs: from discovery to translation. J Intern Med. (2020) 288:518–36. doi: 10.1111/joim.13141, 32686219 PMC7405395

[38] Rubinsztein DC DiFiglia M Heintz N Nixon RA Qin ZH Ravikumar B . Autophagy and its possible roles in nervous system diseases, damage and repair. Autophagy. (2005) 1:11–22. doi: 10.4161/auto.1.1.1513 16874045

[39] Kragh CL Ubhi K Wyss-Coray T Masliah E. Autophagy in dementias. Brain Pathol. (2012) 22:99–109. doi: 10.1111/j.1750-3639.2011.00545.x, 22150925 PMC3243049

[40] Swami M Hendricks AE Gillis T Massood T Mysore J Myers RH . Somatic expansion of the Huntington's disease CAG repeat in the brain is associated with an earlier age of disease onset. Hum Mol Genet. (2009) 18:3039–47. doi: 10.1093/hmg/ddp242, 19465745 PMC2714728

[41] Sarkar S Ravikumar B Rubinsztein DC. Rapamycin and autophagy in Huntington's disease. Autophagy. (2009) 5:538–40.

[42] Ravikumar B Vacher C Berger Z Davies JE Luo S Oroz LG . Inhibition of Mtor induces autophagy and reduces toxicity of Polyglutamine expansions in Fly and mouse models of Huntington disease. Nat Genet. (2004) 36:585–95. doi: 10.1038/ng1362, 15146184

[43] Klomparens EA Ding Y. The neuroprotective mechanisms and effects of Sulforaphane. Brain Circ. (2019) 5:74–83. doi: 10.4103/bc.bc_7_19, 31334360 PMC6611193

[44] Dai S Tang Z Cao J Zhou W Li H Sampson S . Suppression of the Hsf1-mediated Proteotoxic stress response by the metabolic stress sensor AMPK. EMBO J. (2015) 34:275–93. doi: 10.15252/embj.201489062, 25425574 PMC4339117

[45] Anand P Kunnumakkara AB Newman RA Aggarwal BB. Bioavailability of curcumin: problems and promises. Mol Pharm. (2007) 4:807–18. doi: 10.1021/mp700113r, 17999464

[46] Laplante M Sabatini DM. Mtor signaling in growth control and disease. Cell. (2012) 149:274–93. doi: 10.1016/j.cell.2012.03.017, 22500797 PMC3331679

[47] Kim J Guan KL. Mtor as a central hub of nutrient Signalling and cell growth. Nat Cell Biol. (2019) 21:63–71. doi: 10.1038/s41556-018-0205-1, 30602761

[48] Zoncu R Bar-Peled L Efeyan A Wang S Sancak Y Sabatini DM. Mtorc1 senses lysosomal amino acids through an inside-out mechanism that requires the vacuolar H (+)-Atpase. Science. (2011) 334:678–83. doi: 10.1126/science.1207056, 22053050 PMC3211112

[49] Johnson SC Rabinovitch PS Kaeberlein M. Mtor is a key modulator of ageing and age-related disease. Nature. (2013) 493:338–45. doi: 10.1038/nature11861, 23325216 PMC3687363

[50] Bozadjieva N Blandino-Rosano M Chase J Dai XQ Cummings K Gimeno J . Loss of Mtorc1 signaling alters pancreatic Α cell mass and impairs glucagon secretion. J Clin Invest. (2017) 127:4379–93. doi: 10.1172/jci90004, 29106387 PMC5707167

[51] Sena LA Chandel NS. Physiological roles of mitochondrial reactive oxygen species. Mol Cell. (2012) 48:158–67. doi: 10.1016/j.molcel.2012.09.025, 23102266 PMC3484374

[52] Shiloh Y Ziv Y. The Atm protein kinase: regulating the cellular response to genotoxic stress, and more. Nat Rev Mol Cell Biol. (2013) 14:197–210. doi: 10.1038/nrm3546, 23847781

[53] Demidenko ZN Zubova SG Bukreeva EI Pospelov VA Pospelova TV Blagosklonny MV. Rapamycin decelerates cellular senescence. Cell Cycle. (2009) 8:1888–95. doi: 10.4161/cc.8.12.8606, 19471117

[54] Eshraghi M Ahmadi M Afshar S Lorzadeh S Adlimoghaddam A Rezvani Jalal N . Enhancing autophagy in Alzheimer's disease through drug repositioning. Pharmacol Ther. (2022) 237:108171. doi: 10.1016/j.pharmthera.2022.108171, 35304223

[55] Glick D Barth S Macleod KF. Autophagy: cellular and molecular mechanisms. J Pathol. (2010) 221:3–12. doi: 10.1002/path.2697, 20225336 PMC2990190

[56] Stanciu GD Rusu RN Bild V Filipiuc LE Tamba BI Ababei DC. Systemic actions of Sglt2 inhibition on chronic Mtor activation as a shared pathogenic mechanism between Alzheimer's disease and diabetes. Biomedicine. (2021) 9:576. doi: 10.3390/biomedicines9050576, 34069618 PMC8160780

[57] Tan W Qi L Hu X Tan Z. Research Progress in traditional Chinese medicine in the treatment of Alzheimer's disease and related dementias. Front Pharmacol. (2022) 13:921794. doi: 10.3389/fphar.2022.921794, 36506569 PMC9729772

[58] Sun E Motolani A Campos L Lu T. The pivotal role of Nf-kb in the pathogenesis and therapeutics of Alzheimer's disease. Int J Mol Sci. (2022) 23:8972. doi: 10.3390/ijms23168972, 36012242 PMC9408758

[59] Wissler Gerdes EO Zhu Y Weigand BM Tripathi U Burns TC Tchkonia T . Cellular senescence in aging and age-related diseases: implications for neurodegenerative diseases. Int Rev Neurobiol. (2020) 155:203–34. doi: 10.1016/bs.irn.2020.03.019, 32854855 PMC7656525

[60] Zhang W Sun HS Wang X Dumont AS Liu Q. Cellular senescence, DNA damage, and Neuroinflammation in the aging brain. Trends Neurosci. (2024) 47:461–74. doi: 10.1016/j.tins.2024.04.003, 38729785

[61] Tsou YS Lai JH Chen KY Chang CF Huang CC. Therapeutic effect of rapamycin on TDP-43-related pathogenesis in ischemic stroke. Int J Mol Sci. (2022) 24:676. doi: 10.3390/ijms24010676, 36614118 PMC9820757

[62] Spilman P Podlutskaya N Hart MJ Debnath J Gorostiza O Bredesen D . Inhibition of Mtor by rapamycin abolishes cognitive deficits and reduces amyloid-Beta levels in a mouse model of Alzheimer's disease. PLoS One. (2010) 5:e9979. doi: 10.1371/journal.pone.0009979, 20376313 PMC2848616

[63] Shi Q Chang C Saliba A Bhat MA. Microglial Mtor activation upregulates Trem2 and enhances Β-amyloid plaque clearance in the 5xfad Alzheimer's disease model. J Neurosci. (2022) 42:5294–313. doi: 10.1523/jneurosci.2427-21.2022, 35672148 PMC9270922

[64] Hsu CM Lin PM Tsai YT Tsai MS Tseng CH Lin SF . Nvp-Bez235, a dual Pi3k-Mtor inhibitor, suppresses the growth of Fadu Hypopharyngeal squamous cell carcinoma and has a synergistic effect with cisplatin. Cell Death Discov. (2018) 4:57. doi: 10.1038/s41420-018-0060-7, 29760955 PMC5945618

[65] Zhou H Huang S. Role of Mtor signaling in tumor cell motility, invasion and metastasis. Curr Protein Pept Sci. (2011) 12:30–42. doi: 10.2174/138920311795659407, 21190521 PMC3410744

[66] Leontieva OV Blagosklonny MV. Gerosuppression by Pan-Mtor inhibitors. Aging. (2016) 8:3535–51. doi: 10.18632/aging.101155, 28077803 PMC5270685

[67] Lamming DW. Inhibition of the mechanistic target of rapamycin (mTOR)-rapamycin and beyond. Cold Spring Harb Perspect Med. (2016) 6:a025924. doi: 10.1101/cshperspect.a025924, 27048303 PMC4852795

[68] Millichap LE Damiani E Tiano L Hargreaves IP. Targetable pathways for alleviating mitochondrial dysfunction in neurodegeneration of metabolic and non-metabolic diseases. Int J Mol Sci. (2021) 22:22 (21). doi: 10.3390/ijms222111444, 34768878 PMC8583882

[69] Zhao X Fang J Li S Gaur U Xing X Wang H . Artemisinin attenuated hydrogen peroxide (H (2)O (2))-induced oxidative injury in Sh-Sy5y and hippocampal neurons via the activation of Ampk pathway. Int J Mol Sci. (2019) 20:2680. doi: 10.3390/ijms20112680, 31151322 PMC6600327

[70] Zhang Y Ouyang J Zhan L Li Y Li S He Y . Autophagy in homocysteine-induced Huvec senescence. Exp Ther Med. (2023) 26:354. doi: 10.3892/etm.2023.12053, 37324509 PMC10265697

[71] Xiong B Yang C Yang X Luo S Li S Chen C . Arctigenin derivative a-1 ameliorates motor dysfunction and pathological manifestations in Sod1 (G93a) transgenic mice via the Ampk/Sirt1/Pgc-1α and Ampk/Sirt1/Il-1β/Nf-Κb pathways. CNS Neurosci Ther. (2024) 30:e14692. doi: 10.1111/cns.14692, 38872258 PMC11176200

[72] He LL Wang YC Ai YT Wang L Gu SM Wang P . Qiangji decoction alleviates neurodegenerative changes and hippocampal neuron apoptosis induced by D-galactose via regulating Ampk/Sirt1/Nf-Κb signaling pathway. Front Pharmacol. (2021) 12:735812. doi: 10.3389/fphar.2021.735812, 34630111 PMC8495211

[73] Ale Mahmoud Mehraban R Babaei P Rohampour K Jafari A Golipoor Z. Metformin improves memory via AMPK/mTOR-dependent route in a rat model of Alzheimer's disease. Iran J Basic Med Sci. (2024) 27:360–5. doi: 10.22038/ijbms.2023.73075.15879 38333746 PMC10849203

[74] Ou Z Kong X Sun X He X Zhang L Gong Z . Metformin treatment prevents amyloid plaque deposition and memory impairment in app/Ps1 mice. Brain Behav Immun. (2018) 69:351–63. doi: 10.1016/j.bbi.2017.12.009, 29253574

[75] Zhang L Hao J Zheng Y Su R Liao Y Gong X . Fucoidan protects dopaminergic neurons by enhancing the mitochondrial function in a rotenone-induced rat model of Parkinson's disease. Aging Dis. (2018) 9:590–604. doi: 10.14336/ad.2017.0831, 30090649 PMC6065300

[76] Chen C Chen Y Liu T Song D Ma D Cheng O. Dexmedetomidine can enhance Pink1/Parkin-mediated Mitophagy in Mptp-induced Pd mice model by activating Ampk. Oxidative Med Cell Longev. (2022) 2022:7511393. doi: 10.1155/2022/7511393, 35528513 PMC9068320

[77] Ulgherait M Rana A Rera M Graniel J Walker DW. Ampk modulates tissue and organismal aging in a non-cell-autonomous manner. Cell Rep. (2014) 8:1767–80. doi: 10.1016/j.celrep.2014.08.006, 25199830 PMC4177313

[78] Sha X Zou X Liu S Guan C Shi W Gao J . Forkhead box O1 in metabolic dysfunction-associated fatty liver disease: molecular mechanisms and drug research. Front Nutr. (2024) 11:1426780. doi: 10.3389/fnut.2024.1426780, 39021599 PMC11253077

[79] Maiese K. The metabolic basis for nervous system dysfunction in Alzheimer's disease, Parkinson's disease, and Huntington's disease. Curr Neurovasc Res. (2023) 20:314–33. doi: 10.2174/1567202620666230721122957, 37488757 PMC10528135

[80] Jang J Kim SR Lee JE Lee S Son HJ Choe W . Molecular mechanisms of neuroprotection by ketone bodies and ketogenic diet in cerebral ischemia and neurodegenerative diseases. Int J Mol Sci. (2023) 25:124. doi: 10.3390/ijms25010124, 38203294 PMC10779133

[81] Yamamoto M Kensler TW Motohashi H. The Keap1-Nrf2 system: A thiol-based sensor-effector apparatus for maintaining redox homeostasis. Physiol Rev. (2018) 98:1169–203. doi: 10.1152/physrev.00023.2017, 29717933 PMC9762786

[82] McMahon M Itoh K Yamamoto M Hayes JD. Keap1-dependent proteasomal degradation of transcription factor Nrf2 contributes to the negative regulation of antioxidant response element-driven gene expression. J Biol Chem. (2003) 278:21592–600. doi: 10.1074/jbc.M300931200, 12682069

[83] Valko M Leibfritz D Moncol J Cronin MT Mazur M Telser J. Free radicals and antioxidants in normal physiological functions and human disease. Int J Biochem Cell Biol. (2007) 39:44–84. doi: 10.1016/j.biocel.2006.07.001, 16978905

[84] Halliwell B. Free radicals and antioxidants: updating a personal view. Nutr Rev. (2012) 70:257–65. doi: 10.1111/j.1753-4887.2012.00476.x, 22537212

[85] Ammal Kaidery N Ahuja M Thomas B. Crosstalk between Nrf2 signaling and mitochondrial function in Parkinson's disease. Mol Cell Neurosci. (2019) 101:103413. doi: 10.1016/j.mcn.2019.103413, 31644952 PMC6981291

[86] George M Tharakan M Culberson J Reddy AP Reddy PH. Role of Nrf2 in aging, Alzheimer's and other neurodegenerative diseases. Ageing Res Rev. (2022) 82:101756. doi: 10.1016/j.arr.2022.101756, 36243357

[87] Loboda A Damulewicz M Pyza E Jozkowicz A Dulak J. Role of Nrf2/ho-1 system in development, oxidative stress response and diseases: An evolutionarily conserved mechanism. Cell Mol Life Sci. (2016) 73:3221–47. doi: 10.1007/s00018-016-2223-0, 27100828 PMC4967105

[88] Zweig JA Brandes MS Brumbach BH Caruso M Wright KM Quinn JF . Loss of Nrf2 accelerates cognitive decline, exacerbates mitochondrial dysfunction, and is required for the cognitive enhancing effects of *Centella Asiatica* during aging. Neurobiol Aging. (2021) 100:48–58. doi: 10.1016/j.neurobiolaging.2020.11.019, 33486357 PMC7920997

[89] An Y Li Y Hou Y Huang S Pei G. Alzheimer's amyloid-Β accelerates cell senescence and suppresses the Sirt1/Nrf2 pathway in human microglial cells. Oxidative Med Cell Longev. (2022) 2022:3086010. doi: 10.1155/2022/3086010, 36035216 PMC9402294

[90] Tastan B Arioz BI Genc S. Targeting Nlrp3 Inflammasome with Nrf2 inducers in central nervous system disorders. Front Immunol. (2022) 13:865772. doi: 10.3389/fimmu.2022.865772, 35418995 PMC8995746

[91] Zhang C Zhao M Wang B Su Z Guo B Qin L . The Nrf2-Nlrp3-Caspase-1 Axis mediates the neuroprotective effects of Celastrol in Parkinson's disease. Redox Biol. (2021) 47:102134. doi: 10.1016/j.redox.2021.102134, 34600334 PMC8487081

[92] Tang M Ji C Pallo S Rahman I Johnson GVW. Nrf2 mediates the expression of Bag3 and autophagy cargo adaptor proteins and tau clearance in an age-dependent manner. Neurobiol Aging. (2018) 63:128–39. doi: 10.1016/j.neurobiolaging.2017.12.001, 29304346 PMC5801049

[93] Ávila-Román J Quevedo-Tinoco L Oliveros-Ortiz AJ García-Gil S Rodríguez-García G Motilva V . Synthesis and bioevaluation of new stable derivatives of Chrysin-8-C-glucoside that modulate the antioxidant Keap1/Nrf2/ho-1 pathway in human macrophages. Pharmaceuticals (Basel). (2024) 17:1388. doi: 10.3390/ph17101388, 39459027 PMC11510274

[94] Huang L Han Y Zhou Q Sun Z Yan J. Isoliquiritigenin attenuates Neuroinflammation in mice model of Parkinson's disease by promoting Nrf2/Nqo-1 pathway. Transl Neurosci. (2022) 13:301–8. doi: 10.1515/tnsci-2022-0239, 36160039 PMC9468679

[95] Ala M. Sestrin2 signaling pathway regulates Podocyte biology and protects against diabetic nephropathy. J Diabetes Res. (2023) 2023:1–15. doi: 10.1155/2023/8776878, 36818747 PMC9937769

[96] Wang L Zhang X Xiong X Zhu H Chen R Zhang S . Nrf2 regulates oxidative stress and its role in cerebral ischemic stroke. Antioxidants. (2022) 11:2377. doi: 10.3390/antiox11122377, 36552584 PMC9774301

[97] Cacabelos R Carril JC Cacabelos N Kazantsev AG Vostrov AV Corzo L . Sirtuins in Alzheimer's disease: Sirt2-related Genophenotypes and implications for Pharmacoepigenetics. Int J Mol Sci. (2019) 20:1249. doi: 10.3390/ijms20051249, 30871086 PMC6429449

[98] Lu P Han D Zhu K Jin M Mei X Lu H. Effects of Sirtuin 1 on microglia in spinal cord injury: involvement of Wnt/Β-catenin signaling pathway. Neuroreport. (2019) 30:867–74. doi: 10.1097/wnr.0000000000001293, 31373965

[99] de Oliveira RM Vicente Miranda H Francelle L Pinho R Szegö ÉM Martinho R . The mechanism of Sirtuin 2-mediated exacerbation of alpha-Synuclein toxicity in models of Parkinson disease. PLoS Biol. (2017) 15:e2000374. doi: 10.1371/journal.pbio.2000374, 28257421 PMC5336201

[100] Shen Y Wang X Nan N Fu X Zeng R Yang Y . Sirt3-mediated deacetylation of Sdha rescues mitochondrial bioenergetics contributing to neuroprotection in rotenone-induced Pd models. Mol Neurobiol. (2024) 61:4402–20. doi: 10.1007/s12035-023-03830-w, 38087172

[101] Park JH Burgess JD Faroqi AH DeMeo NN Fiesel FC Springer W . Alpha-synuclein-induced mitochondrial dysfunction is mediated via a Sirtuin 3-dependent pathway. Mol Neurodegener. (2020) 15:5. doi: 10.1186/s13024-019-0349-x, 31931835 PMC6956494

[102] Geng A Sun J Tang H Yu Y Wang X Zhang J . Sirt2 Promotes Base excision repair by transcriptionally activating Ogg1 in an Atm/Atr-dependent manner. Nucleic Acids Res. (2024) 52:5107–20. doi: 10.1093/nar/gkae190, 38554113 PMC11109957

[103] Sherin F Gomathy S Antony S. Sirtuin3 in Neurological Disorders. Curr Drug Res Rev. (2021) 13:140–7. doi: 10.2174/2589977512666201207200626, 33290206

[104] Wang R Wu Y Liu R Liu M Li Q Ba Y . Deciphering therapeutic options for neurodegenerative diseases: insights from Sirt1. J Mol Med (Berl). (2022) 100:537–53. doi: 10.1007/s00109-022-02187-2, 35275221

[105] Akter R Afrose A Rahman MR Chowdhury R Nirzhor SSR Khan RI . A comprehensive analysis into the therapeutic application of natural products as Sirt6 modulators in Alzheimer's disease, aging, cancer, inflammation, and diabetes. Int J Mol Sci. (2021) 22:4180. doi: 10.3390/ijms22084180, 33920726 PMC8073883

[106] Cong L Ma J Zhang Y Zhou Y Cong X Hao M. Effect of anti-skin disorders of Ginsenosides- a systematic review. J Ginseng Res. (2023) 47:605–14. doi: 10.1016/j.jgr.2023.04.005, 37720567 PMC10499590

[107] Panossian A Efferth T. Network pharmacology of Adaptogens in the assessment of their pleiotropic therapeutic activity. Pharmaceuticals. (2022) 15:1051. doi: 10.3390/ph15091051, 36145272 PMC9504187

[108] Han Y Liu D Cheng Y Ji Q Liu M Zhang B . Maintenance of mitochondrial homeostasis for Alzheimer's disease: strategies and challenges. Redox Biol. (2023) 63:102734. doi: 10.1016/j.redox.2023.102734, 37159984 PMC10189488

[109] Huang L Liu LF Liu J Dou L Wang GY Liu XQ . Ginsenoside Rg1 protects against neurodegeneration by inducing neurite outgrowth in cultured hippocampal neurons. Neural Regen Res. (2016) 11:319–25. doi: 10.4103/1673-5374.177741, 27073387 PMC4810998

[110] Liu J He J Huang L Dou L Wu S Yuan Q. Neuroprotective effects of Ginsenoside Rb1 on hippocampal neuronal injury and neurite outgrowth. Neural Regen Res. (2014) 9:943–50. doi: 10.4103/1673-5374.133137, 25206916 PMC4146219

[111] Hou J Kim S Sung C Choi C. Ginsenoside Rg3 prevents oxidative stress-induced astrocytic senescence and ameliorates senescence paracrine effects on glioblastoma. Molecules. (2017) 22:22 (9). doi: 10.3390/molecules22091516, 28891967 PMC6151485

[112] Hou J Yun Y Jeon B Baek J Kim S. Ginsenoside F1-mediated telomere preservation delays cellular senescence. Int J Mol Sci. (2023) 24:14241. doi: 10.3390/ijms241814241, 37762556 PMC10531559

[113] Yang KE Nam SB Jang M Park J Lee GE Cho YY . Ginsenoside Rb2 suppresses cellular senescence of human dermal fibroblasts by inducing autophagy. J Ginseng Res. (2023) 47:337–46. doi: 10.1016/j.jgr.2022.11.004, 36926607 PMC10014224

[114] Hou JG Jeon BM Yun YJ Cui CH Kim SC. Ginsenoside Rh2 ameliorates doxorubicin-induced senescence bystander effect in breast carcinoma cell Mda-Mb-231 and Normal epithelial cell Mcf-10a. Int J Mol Sci. (2019) 20:1244. doi: 10.3390/ijms20051244, 30871042 PMC6429443

[115] Zhang X Wang Y Ma C Yan Y Yang Y Wang X . Ginsenoside Rd and ginsenoside re offer neuroprotection in a novel model of Parkinson's disease. Am J Neurodegener Dis. (2016) 5:52–61. 27073742 PMC4788731

[116] Oh SJ Kim K Lim CJ. Protective properties of ginsenoside Rb1 against UV-B radiation-induced oxidative stress in human dermal keratinocytes. Pharmazie. (2015) 70:381–7. doi: 10.31083/ph.2015.4884, 26189299

[117] Zhou S Gao Y Jiang W Huang M Xu A Paxton JW. Interactions of herbs with cytochrome P450. Drug Metab Rev. (2003) 35:35–98. doi: 10.1081/dmr-120018248, 12635815

[118] Di Meo F Cuciniello R Margarucci S Bergamo P Petillo O Peluso G . Ginkgo biloba prevents oxidative stress-induced apoptosis blocking P53 activation in neuroblastoma cells. Antioxidants. (2020) 9:279. doi: 10.3390/antiox9040279, 32224984 PMC7222193

[119] Wang K Ni J Zhu X Zhu L Li Y Zhou F. Ginkgo biloba extract protects human Neuroblastoma Sh-Sy5y cells against oxidative glutamate toxicity by activating Redoxosome-P66shc. Exp Ther Med. (2021) 22:951. doi: 10.3892/etm.2021.10383, 34335893 PMC8290427

[120] Zheng Y Wu Z Yi F Orange M Yao M Yang B . By activating Akt/Enos Bilobalide B inhibits autophagy and promotes angiogenesis following focal cerebral ischemia reperfusion. Cell Physiol Biochem. (2018) 47:604–16. doi: 10.1159/000490016, 29794436

[121] Adekeye AO Fafure AA Omodele MM Adedayo LD Ekundina VO Adekomi DA . Flavonoid glycoside fraction of ginkgo biloba extract modulates antioxidants imbalance in vanadium-induced brain damage. AIMS Neurosci. (2023) 10:178–89. doi: 10.3934/Neuroscience.2023015, 37426781 PMC10323262

[122] Liu Y Ye J Fan Z Wu X Zhang Y Yang R . Ginkgetin alleviates inflammation and senescence by targeting sting. Adv Sci. (2024) 12:e2407222. doi: 10.1002/advs.202407222, 39558862 PMC11727237

[123] Al Kury LT Dayyan F Ali Shah F Malik Z Khalil AAK Alattar A . Ginkgo biloba extract protects against methotrexate-induced hepatotoxicity: a computational and pharmacological approach. Molecules. (2020) 25:2540. doi: 10.3390/molecules25112540, 32486047 PMC7321289

[124] Wang Z Zhang P Wang Q Sheng X Zhang J Lu X . Protective effects of ginkgo biloba dropping pills against liver ischemia/reperfusion injury in mice. Chin Med. (2020) 15:122. doi: 10.1186/s13020-020-00404-z, 33292377 PMC7678318

[125] Ge W Ren C Xing L Guan L Zhang C Sun X . Ginkgo biloba extract improves cognitive function and increases neurogenesis by reducing aβ pathology in 5×fad mice. Am J Transl Res. (2021) 13:1471–82. 33841671 PMC8014356

[126] Zhang GJ Zheng D Yu H Luo XP Wu W. Ginkgo biloba extract ameliorates scopolamine-induced memory deficits via rescuing synaptic damage. Curr Med Sci. (2022) 42:474–82. doi: 10.1007/s11596-022-2582-8, 35678907

[127] Gorantla NV Das R Chidambaram H Dubey T Mulani FA Thulasiram HV . Basic limonoid modulates chaperone-mediated proteostasis and dissolve tau fibrils. Sci Rep. (2020) 10:4023. doi: 10.1038/s41598-020-60773-1, 32132570 PMC7055235

[128] Ma W Wei S Peng W Sun T Huang J Yu R . Antioxidant effect of Polygonatum Sibiricum polysaccharides in D-galactose-induced heart aging mice. Biomed Res Int. (2021) 2021:6688855. doi: 10.1155/2021/6688855, 33860051 PMC8024086

[129] Lee SY Han SY Shim YJ Han JJ Cho D Kim JE . Effect of ginkgo biloba extract on N-methyl-D-aspartic acid receptor subunit 2b expression in a salicylate-induced ototoxicity model. Clin Exp Otorhinolaryngol. (2019) 12:169–75. doi: 10.21053/ceo.2018.00983, 30360042 PMC6453795

[130] Liu Q Li X Luo Y. Tanshinone Iia delays liver aging by modulating oxidative stress. Front Pharmacol. (2024) 15:1434024. doi: 10.3389/fphar.2024.1434024, 39415831 PMC11480062

[131] Li H Han W Wang H Ding F Xiao L Shi R . Tanshinone Iia inhibits glutamate-induced oxidative toxicity through prevention of mitochondrial dysfunction and suppression of Mapk activation in Sh-Sy5y human Neuroblastoma cells. Oxidative Med Cell Longev. (2017) 2017:4517486. doi: 10.1155/2017/4517486, 28690763 PMC5485345

[132] Luo M Cai X Yan D Liu X Guo SW. Sodium Tanshinone Iia sulfonate restrains Fibrogenesis through induction of senescence in mice with induced deep endometriosis. Reprod Biomed Online. (2020) 41:373–84. doi: 10.1016/j.rbmo.2020.04.006, 32651107

[133] Banerjee J Das A Sinha M Saha S. Biological efficacy of medicinal plant extracts in preventing oxidative damage. Oxidative Med Cell Longev. (2018) 2018:7904349. doi: 10.1155/2018/7904349, 30302174 PMC6158943

[134] Wei W Heng YY Wu FF Dong HY Zhang PF Li JX . Sodium Tanshinone Iia sulfonate alleviates vascular senescence in diabetic mice by modulating the A20-Nfκb-Nlrp3 Inflammasome-catalase pathway. Sci Rep. (2024) 14:17665. doi: 10.1038/s41598-024-68169-1, 39085294 PMC11291694

[135] Jiang C Zhu W Yan X Shao Q Xu B Zhang M . Rescue therapy with Tanshinone Iia hinders transition of acute kidney injury to chronic kidney disease via targeting Gsk3β. Sci Rep. (2016) 6:36698. doi: 10.1038/srep36698, 27857162 PMC5114614

[136] Wu J Li M Li A Ji X. Mechanism of *Salvia miltiorrhiza* Bge. For the treatment of ischemic stroke based on bioinformatics and network pharmacology. Evid Based Complement Alternat Med. (2022) 2022:1–11. doi: 10.1155/2022/1767421, 36133785 PMC9484879

[137] Wang T Zhang X Liu W Ning F Hu X Qin L . Identification of diagnostic molecules and potential traditional Chinese medicine components for Alzheimer's disease by single cell RNA sequencing combined with a systematic framework for network pharmacology. Front Med. (2023) 10:1335512. doi: 10.3389/fmed.2023.1335512 PMC10799563 38249960

[138] Hu G Liu H Wang M Peng W. Iq motif containing Gtpase-activating protein 3 (Iqgap3) inhibits Kaempferol-induced apoptosis in breast Cancer cells by extracellular signal-regulated kinases 1/2 (Erk1/2) signaling activation. Med Sci Monit. (2019) 25:7666–74. doi: 10.12659/msm.915642, 31605603 PMC6807529

[139] Xiao X Xu M Fang Y. Tanshinone Iia attenuates hydrogen peroxide-induced senescence of human umbilical vein endothelial cells through activating Sirt1/Enos pathway. Xi Bao Yu Fen Zi Mian Yi Xue Za Zhi. (2019) 35:806–11. 31750822

[140] Chai R Ye Z Xue W Shi S Wei Y Hu Y . Tanshinone Iia inhibits Cardiomyocyte Pyroptosis through Tlr4/Nf-Κb P65 pathway after acute myocardial infarction. Front Cell Dev Biol. (2023) 11:1252942. doi: 10.3389/fcell.2023.1252942, 37766966 PMC10520722

[141] Ji G Li Y Zhang Z Li H Sun P. Recent advances of novel targeted drug delivery systems based on natural medicine monomers against hepatocellular carcinoma. Heliyon. (2024) 10:e24667. doi: 10.1016/j.heliyon.2024.e24667, 38312669 PMC10834828

[142] Ma Z Wu Y Xu J Cao H Du M Jiang H . Sodium tanshinone Iia sulfonate ameliorates oxygen-glucose deprivation/reoxygenation-induced neuronal injury via protection of mitochondria and promotion of autophagy. Neurochem Res. (2023) 48:3378–90. doi: 10.1007/s11064-023-03985-x, 37436612

[143] Meng Z Meng L Wang K Li J Cao X Wu J . Enhanced hepatic targeting, biodistribution and Antifibrotic efficacy of Tanshinone Iia loaded globin nanoparticles. Eur J Pharm Sci. (2015) 73:35–43. doi: 10.1016/j.ejps.2015.03.002, 25769523

[144] Qi B Ji Q Wen Y Liu L Guo X Hou G . *Lycium Barbarum* polysaccharides protect human Lens epithelial cells against oxidative stress-induced apoptosis and senescence. PLoS One. (2014) 9:e110275. doi: 10.1371/journal.pone.0110275, 25333784 PMC4198253

[145] Fang F Peng T Yang S Wang W Zhang Y Li H. *Lycium Barbarum* polysaccharide attenuates the cytotoxicity of mutant huntingtin and increases the activity of Akt. Int J Dev Neurosci. (2016) 52:66–74. doi: 10.1016/j.ijdevneu.2016.05.004, 27196502

[146] Zhao P Ma NT Chang RY Li YX Hao YJ Yang WL . Mechanism of *Lycium Barbarum* polysaccharides on primary cultured rat hippocampal neurons. Cell Tissue Res. (2017) 369:455–65. doi: 10.1007/s00441-017-2648-2, 28656471

[147] Xia G Xin N Liu W Yao H Hou Y Qi J. Inhibitory effect of *Lycium Barbarum* polysaccharides on cell apoptosis and senescence is potentially mediated by the P53 signaling pathway. Mol Med Rep. (2014) 9:1237–41. doi: 10.3892/mmr.2014.1964, 24549741

[148] Zheng H Liang X Zhou H Zhou T Liu X Duan J . Integrated gut microbiota and fecal metabolome analyses of the effect of *Lycium Barbarum* polysaccharide on D-galactose-induced premature ovarian insufficiency. Food Funct. (2023) 14:7209–21. doi: 10.1039/d3fo01659e, 37463025

[149] Zheng X Wang J Bi F Li Y Xiao J Chai Z . Protective effects of *Lycium barbarum* polysaccharide on ovariectomy-induced cognition reduction in aging mice. Int J Mol Med. (2021) 48:48 (1). doi: 10.3892/ijmm.2021.4954, 33955518 PMC8121556

[150] Li C Shi S. Neuroprotective effect of Huperzine a on D-galactose-induced hearing dysfunction. Ear Nose Throat J. (2021) 100:269s–76s. doi: 10.1177/0145561319864570, 31554431

[151] Zhang Y Huang W Tian Q Bai G Wu W Yin H . Network pharmacology and biochemical experiments reveal the Antiapoptotic mechanism of Huperzine a for treating diabetic retinopathy. Br J Ophthalmol. (2024) 108:989–98. doi: 10.1136/bjo-2023-323639, 37339867

[152] Shi J Chen W Tang J Zhang C Qi M Zheng X . Huperzine a protected against Ferroptosis via activating Pi3k/Akt signaling in lipopolysaccharide induced acute lung injury. Eur J Pharmacol. (2024) 983:177004. doi: 10.1016/j.ejphar.2024.177004, 39278310

[153] Kong YR Tay KC Su YX Wong CK Tan WN Khaw KY. Potential of naturally derived alkaloids as multi-targeted therapeutic agents for neurodegenerative diseases. Molecules. (2021) 26:26 (3). doi: 10.3390/molecules26030728, 33573300 PMC7866829

[154] Mak S Li W Fu H Luo J Cui W Hu S . Promising Tacrine/Huperzine a-based dimeric acetylcholinesterase inhibitors for neurodegenerative disorders: from relieving symptoms to modifying diseases through multitarget. J Neurochem. (2021) 158:1381–93. doi: 10.1111/jnc.15379, 33930191 PMC8458250

[155] Lu H Jiang M Lu L Zheng G Dong Q. Ultrastructural mitochondria changes in Perihematomal brain and neuroprotective effects of Huperzine a after acute intracerebral hemorrhage. Neuropsychiatr Dis Treat. (2015) 11:2649–57. doi: 10.2147/ndt.S92158, 26508860 PMC4610774

[156] Shoaib S Ansari MA Fatease AA Safhi AY Hani U Jahan R . Plant-derived bioactive compounds in the Management of Neurodegenerative Disorders: challenges, future directions and molecular mechanisms involved in neuroprotection. Pharmaceutics. (2023) 15:15 (3). doi: 10.3390/pharmaceutics15030749, 36986610 PMC10057544

[157] Zhang HY Yan H Tang XC. Non-cholinergic effects of Huperzine A: beyond inhibition of acetylcholinesterase. Cell Mol Neurobiol. (2008) 28:173–83. doi: 10.1007/s10571-007-9163-z, 17657601 PMC11516533

[158] Lv H Wang Y Yang X Ling G Zhang P. Application of curcumin nanoformulations in Alzheimer's disease: prevention, diagnosis and treatment. Nutr Neurosci. (2023) 26:727–42. doi: 10.1080/1028415x.2022.2084550, 35694842

[159] Assi AA Farrag MMY Badary DM Allam EAH Nicola MA. Protective effects of curcumin and ginkgo biloba extract combination on A new model of Alzheimer's disease. Inflammopharmacology. (2023) 31:1449–64. doi: 10.1007/s10787-023-01164-6, 36856916 PMC10229698

[160] Bielak-Zmijewska A Grabowska W Ciolko A Bojko A Mosieniak G Bijoch Ł . The role of curcumin in the modulation of ageing. Int J Mol Sci. (2019) 20:1239. doi: 10.3390/ijms20051239, 30871021 PMC6429134

[161] Singh A Yadawa AK Rizvi SI. Curcumin protects against aging-related stress and dysfunction through autophagy activation in rat brain. Mol Biol Rep. (2024) 51:694. doi: 10.1007/s11033-024-09639-7, 38796662

[162] Grabowska W Sikora E Bielak-Zmijewska A. Sirtuins, a promising target in slowing down the ageing process. Biogerontology. (2017) 18:447–76. doi: 10.1007/s10522-017-9685-9, 28258519 PMC5514220

[163] Azami SH Nazarian H Abdollahifar MA Eini F Farsani MA Novin MG. The antioxidant curcumin postpones ovarian aging in young and middle-aged mice. Reprod Fertil Dev. (2020) 32:292–303. doi: 10.1071/rd18472, 31656219

[164] Xu D Li L Yu Z. Effect and mechanism of curcumin on Colon Cancer cell senescence through early growth response 1 (Egr1). Transl Cancer Res. (2024) 13:3251–61. doi: 10.21037/tcr-24-26, 39145095 PMC11319940

[165] Zhang J Zheng Y Luo Y Du Y Zhang X Fu J. Curcumin inhibits Lps-induced Neuroinflammation by promoting microglial M2 polarization via Trem2/ Tlr4/ Nf-Κb pathways in Bv2 cells. Mol Immunol. (2019) 116:29–37. doi: 10.1016/j.molimm.2019.09.020, 31590042

[166] Abdul Manap AS Wei Tan AC Leong WH Yin Chia AY Vijayabalan S Arya A . Synergistic effects of curcumin and Piperine as potent acetylcholine and Amyloidogenic inhibitors with significant neuroprotective activity in Sh-Sy5y cells via computational molecular modeling and in vitro assay. Front Aging Neurosci. (2019) 11:206. doi: 10.3389/fnagi.2019.00206, 31507403 PMC6718453

[167] Ahmed T Enam SA Gilani AH. Curcuminoids enhance memory in an amyloid-infused rat model of Alzheimer's disease. Neuroscience. (2010) 169:1296–306. doi: 10.1016/j.neuroscience.2010.05.078, 20538041

[168] Barbara R Belletti D Pederzoli F Masoni M Keller J Ballestrazzi A . Novel curcumin loaded nanoparticles engineered for blood-brain barrier crossing and able to disrupt Abeta aggregates. Int J Pharm. (2017) 526:413–24. doi: 10.1016/j.ijpharm.2017.05.015, 28495580

[169] Galzigna L Rizzoli V Schiavo R Ferro L Vittadini G. Denaturing effect of phenolic compounds on proteins. Arch Biochem Biophys. (1972) 149:222–8.4335960

[170] Lefranc F Sauvage S Van Goietsenoven G Hovey BM Pirotte S De Caro M . Curcumin and quercetin at high concentrations: friend or foe for proteins? Molecules. (2019) 24:1920. doi: 10.3390/molecules24101920 31109069 PMC6571768

[171] Masuda M Suzuki N Taniguchi S Oikawa T Nonaka T Iwatsubo T . Inhibition of alpha-synuclein fibril assembly by small molecules. Biochemistry. (2006) 45:6085–94. 16681381 10.1021/bi0600749

[172] Ono K Yamada M. Antioxidant compounds have potent anti-fibrillogenic and fibril-destabilizing effects for alpha-synuclein fibrils in vitro. J Neurochem. (2006) 97:105–15. doi: 10.1111/j.1471-4159.2006.03692.x, 16524383

[173] Zoico E Nori N Darra E Tebon M Rizzatti V Policastro G . Senolytic effects of quercetin in an in vitro model of pre-adipocytes and adipocytes induced senescence. Sci Rep. (2021) 11:23237. doi: 10.1038/s41598-021-02544-0, 34853352 PMC8636588

[174] Jiang YH Jiang LY Wang YC Ma DF Li X. Quercetin attenuates atherosclerosis via modulating oxidized Ldl-induced endothelial cellular senescence. Front Pharmacol. (2020) 11:512. doi: 10.3389/fphar.2020.00512, 32410992 PMC7198817

[175] Wu W Wu X Qiu L Wan R Zhu X Chen S . Quercetin influences intestinal Dysbacteriosis and delays alveolar epithelial cell senescence by regulating Pten/Pi3k/Akt signaling in pulmonary fibrosis. Naunyn Schmiedeberg's Arch Pharmacol. (2024) 397:4809–22. doi: 10.1007/s00210-023-02913-8, 38153514 PMC11166760

[176] Kim SG Sung JY Kim JR Choi HC. Quercetin-induced apoptosis ameliorates vascular smooth muscle cell senescence through amp-activated protein kinase signaling pathway. Korean J Physiol Pharmacol. (2020) 24:69–79. doi: 10.4196/kjpp.2020.24.1.69, 31908576 PMC6940493

[177] Ding K Jiang W Zhan W Xiong C Chen J Wang Y . The therapeutic potential of quercetin for cigarette smoking-induced chronic obstructive pulmonary disease: A narrative review. Ther Adv Respir Dis. (2023) 17:17534666231170800. doi: 10.1177/17534666231170800, 37154390 PMC10170608

[178] Chiang MC Tsai TY Wang CJ. The potential benefits of quercetin for brain health: A review of anti-inflammatory and neuroprotective mechanisms. Int J Mol Sci. (2023) 24:24 (7). doi: 10.3390/ijms24076328, 37047299 PMC10094159

[179] Childs BG Baker DJ Kirkland JL Campisi J van Deursen JM. Senescence and apoptosis: dueling or complementary cell fates? EMBO Rep. (2014) 15:1139–53. doi: 10.15252/embr.201439245, 25312810 PMC4253488

[180] Zhu Y Tchkonia T Pirtskhalava T Gower AC Ding H Giorgadze N . The Achilles' heel of senescent cells: from transcriptome to senolytic drugs. Aging Cell. (2015) 14:644–58. doi: 10.1111/acel.12344, 25754370 PMC4531078

[181] Hickson LJ Langhi Prata LGP Bobart SA Evans TK Giorgadze N Hashmi SK . Senolytics decrease senescent cells in humans: preliminary report from a clinical trial of dasatinib plus quercetin in individuals with diabetic kidney disease. EBioMedicine. (2019) 47:446–56. doi: 10.1016/j.ebiom.2019.08.069, 31542391 PMC6796530

[182] Kilic Eren M Kilincli A Eren Ö. Resveratrol induced premature senescence is associated with DNA damage mediated Sirt1 and Sirt2 down-regulation. PLoS One. (2015) 10:e0124837. doi: 10.1371/journal.pone.0124837, 25924011 PMC4414559

[183] Alayev A Doubleday PF Berger SM Ballif BA Holz MK. Phosphoproteomics reveals resveratrol-dependent inhibition of Akt/Mtorc1/S6k1 signaling. J Proteome Res. (2014) 13:5734–42. doi: 10.1021/pr500714a, 25311616 PMC4258159

[184] Ido Y Duranton A Lan F Weikel KA Breton L Ruderman NB. Resveratrol prevents oxidative stress-induced senescence and proliferative dysfunction by activating the Ampk-Foxo3 Cascade in cultured primary human keratinocytes. PLoS One. (2015) 10:e0115341. doi: 10.1371/journal.pone.0115341, 25647160 PMC4315597

[185] Zeng XL Yang XN Liu XJ. Resveratrol attenuates cigarette smoke extract induced cellular senescence in human airway epithelial cells by regulating the Mir-34a/Sirt1/Nf-Κb pathway. Medicine. (2022) 101:e31944. doi: 10.1097/md.0000000000031944, 36401446 PMC9678562

[186] Shaito A Posadino AM Younes N Hasan H Halabi S Alhabbash D . Potential adverse effects of resveratrol: a literature review. Int J Mol Sci. (2020) 21:2084. doi: 10.3390/ijms21062084 32197410 PMC7139620

[187] Walle T Hsieh F DeLegge MH Oatis JE Jr Walle UK. High absorption but very low bioavailability of oral resveratrol in humans. Drug Metab Dispos. (2004) 32:1371–7. 10.1124/dmd.104.000885 15333514

[188] Begley DJ. Delivery of therapeutic agents to the central nervous system: the problems and the possibilities. Pharmacol Ther. (2004) 104:29–45. doi: 10.1016/j.pharmthera.2004.08.001, 15500907

[189] Cardona F Andrés-Lacueva C Tulipani S Tinahones FJ Queipo-Ortuño MI. Benefits of polyphenols on gut microbiota and implications in human health. J Nutr Biochem. (2013) 24:1415–22. doi: 10.1016/j.jnutbio.2013.05.001, 23849454

[190] Ogrodnik M Evans SA Fielder E Victorelli S Kruger P Salmonowicz H . Whole-body senescent cell clearance alleviates age-related brain inflammation and cognitive impairment in mice. Aging Cell. (2021) 20:e13296. doi: 10.1111/acel.13296, 33470505 PMC7884042

[191] Dobrovolskaia MA Aggarwal P Hall JB McNeil SE. Preclinical studies to understand nanoparticle interaction with the immune system and its application in in vivo nanoparticle safety evaluation. Br J Pharmacol. (2007) 151:478–85. doi: 10.1038/sj.bjp.0703586

[192] Dobrovolskaia MA McNeil SE. Immunological properties of engineered nanomaterials. Nat Nanotechnol. (2007) 2:469–78. doi: 10.1038/nnano.2007.223 18654343

[193] Xu M Pirtskhalava T Farr JN Weigand BM Palmer AK Weivoda MM . Senolytics improve physical function and increase lifespan in old age. Nat Med. (2018) 24:1246–56. doi: 10.1038/s41591-018-0092-9, 29988130 PMC6082705

[194] Tong Y Zhang Z Wang S. Role of mitochondria in retinal pigment epithelial aging and degeneration. Front Aging. (2022) 3:926627. doi: 10.3389/fragi.2022.926627, 35912040 PMC9337215

[195] Lin RR Jin LL Xue YY Zhang ZS Huang HF Chen DF . Hybrid membrane-coated nanoparticles for precise targeting and synergistic therapy in Alzheimer's disease. Adv Sci. (2024) 11:e2306675. doi: 10.1002/advs.202306675, 38647399 PMC11200089

[196] Feng Q Zhang X Zhao X Liu J Wang Q Yao Y . Intranasal delivery of pure nanodrug loaded liposomes for Alzheimer's disease treatment by efficiently regulating microglial polarization. Small. (2024) 20:e2405781. doi: 10.1002/smll.202405781, 39370581

[197] Lei T Yang Z Jiang C Wang X Yang W Yang X . Mannose-integrated nanoparticle hitchhike glucose transporter 1 recycling to overcome various barriers of Oral delivery for Alzheimer's disease therapy. ACS Nano. (2024) 18:3234–50. doi: 10.1021/acsnano.3c09715, 38214975

[198] Liu J Liu C Zhang J Zhang Y Liu K Song JX . A self-assembled Α-Synuclein Nanoscavenger for Parkinson's disease. ACS Nano. (2020) 14:1533–49. doi: 10.1021/acsnano.9b06453, 32027482

[199] Zhen JB Yi J Ding HH Yang KW. Self-assembled cationic nanoparticles combined with curcumin against multidrug-resistant Bacteria. ACS Omega. (2022) 7:29909–22. doi: 10.1021/acsomega.2c02855, 36061679 PMC9434756

[200] Campisi J Kapahi P Lithgow GJ Melov S Newman JC Verdin E. From discoveries in ageing research to therapeutics for healthy ageing. Nature. (2019) 571:183–92. doi: 10.1038/s41586-019-1365-2, 31292558 PMC7205183

